# Activating Lithium Titanate for High-Performance and Stable Electrochemical Direct Lithium Extraction

**DOI:** 10.1021/acs.est.5c14846

**Published:** 2026-01-16

**Authors:** Bing Zhao, Longqian Xu, Yingjun Qiao, Zhiqiang Qian, Wenfei Wei, Xudong Zhang, Zhong Liu, Shihong Lin

**Affiliations:** † Key Laboratory of Green and High-End Utilization of Salt Lake Resources, Qinghai Province Key Laboratory of Resources and Chemistry of Salt Lakes, 74779 Qinghai Institute of Salt Lakes, Chinese Academy of Sciences, Xining, Qinghai 810008, China; ‡ Department of Civil and Environmental Engineering, 5718 Vanderbilt University, Nashville, Tennessee 37235-1831, United States; § Department of Chemical and Biomolecular Engineering, Vanderbilt University, Nashville, Tennessee 37235-1604, United States

**Keywords:** direct lithium extraction, spinel lithium titanate, site-selective Mn doping, electrosorption, redox activation, sustainable resource recovery

## Abstract

Direct lithium extraction (DLE) from brine using electrosorption processes offers an environmentally sustainable alternative to evaporation-based mining, but its advancement has been hindered by the lack of electrode materials that combine high selectivity, fast kinetics, and long-term stability. Lithium titanate (LTO) is structurally robust and chemically selective yet remains electrochemically inactive under aqueous conditions. In contrast, manganese-based spinels exhibit strong redox activity but suffer from structural degradation. Here, we report a site-selective doping strategy that integrates the strengths of both material classes by incorporating Mn into the spinel framework of LTO. Crystallographic, spectroscopic, and electrochemical analyses reveal that Mn substitution of specific lattice sites modulates both Li^+^ transport pathways and redox-active centers, enabling enhanced intercalation kinetics and electronic conductivity without compromising the structural integrity of the crystal lattice. The optimized electrode material (H_1_._33_Ti_1_._17_Mn_0_._5_O_4_) achieves a record Li^+^ adsorption capacity of 43.58 mg/g at 350 ppm of Li^+^ with high selectivity and minimal capacity loss after repeated cycling with real salt lake brine. This approach transforms an electrochemically inert but stable spinel into a redox-active host, providing a generalizable pathway for designing acid-free and energy-efficient electrodes for sustainable lithium recovery from complex saline resources.

## Graphical abstract

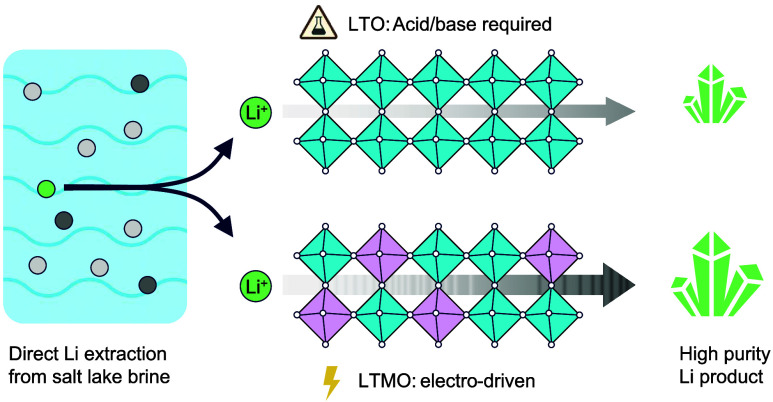

## 1 Introduction

The accelerating electrification of the transportation and energy storage sectors has sharply increased the global demand for lithium. Direct lithium extraction (DLE) from brines has emerged as a sustainable alternative to evaporation-based processes, yet its progress remains limited by the lack of robust and selective electrode materials.^1^ Li can be mined from ores or brines, with the latter currently being the more cost-effective approach that accounts for 62% of the global Li production.^2^ Existing Li mining from brines requires extended pond evaporation to adjust the brine chemistry to become favorable for the subsequent Li purification and precipitation.^3^ However, DLE (a category of Li extraction processes without pond evaporation) has recently gained considerable interest in research and development. DLE can unlock new Li brine resources in regions where conventional Li extraction based on pond evaporation is nonviable due to compositional, climatic, and regulatory constraints.^4^

Among various core unit processes for DLE, adsorption and electrosorption are two promising and closely related technological platforms. Adsorption uses Li-selective adsorbents such as lithium–aluminum layer double hydroxide (LiAl-LDH), lithium titanate (LTO), and lithium manganese oxide (LMO).^5^ The desorption step involves washing adsorbents with strong acids (as with LTO and LMO) or a large volume of freshwater (as with LiAl-LDH), which is a critical constraint in brine source regions that are environmentally sensitive and/or have limited access to freshwater. Electrosorption addresses this challenge by using electrochemical driving force to enable the desorption of Li ions to a relatively small volume of eluate without using strong acids.^6^ The two most common classes of electrodes for electrosorption-based Li extraction are LMO and lithium iron phosphate (LFP).^7^ Both LMO- or LFP-based electrodes face the challenge of long-term material stability due to dissolution of electrode materials and/or irreversible intercalation of competing cations. A recent analysis has shown that even the most stable LMO and LFP electrodes lost >8% of the initial capacity after 20 cycles.^8^

Spinel-structured LTO is known for its exceptional stability, outstanding selectivity, and high equilibrium adsorption capacity. The reported adsorption capacity of LTO is 10–30 mg of Li per gram of adsorbent (mg/g), which is substantially larger than the reported capacity of the most widely used LiAl-LDH-based adsorbent (2–10 mg/g). However, the practical application of LTO is hindered by its slow adsorption kinetics under neutral or acidic conditions, typically requiring multiple or even tens of hours to reach saturation without pH adjustment.^9^ While alkaline conditions (e.g., pH > 10) can enhance LTO’s adsorption rate, adjusting the pH of a large volume of low-Li concentration brines is economically impractical due to the excessive base consumption. A promising strategy to overcome the kinetic limitation of LTO without excessive use of chemicals is to use electrochemical driving force for adsorption and desorption. In principle, electrosorption can promote fast Li uptake to LTO without a high-pH environment and enable the release of Li from saturated LTO without using strong acids (Figure 1). In other words, the coupling of electrosorption and LTO can solve their respective problems: LTO can address the stability challenge of conventional electrode materials (LMO and LFP) for electrosorption, whereas the use of electrosorption can address the limitation of LTO of requiring excessive base (for adsorption) and strong acids (for desorption).^10^ Unfortunately, while LTO has been used as an electrode material in batteries at higher voltage, it is not electroactive in water within the voltage window to avoid electrolysis. Therefore, LTO in its pure form cannot be used as an effective electrode for electrosorption-based Li extraction.^11^

**Figure 1:**
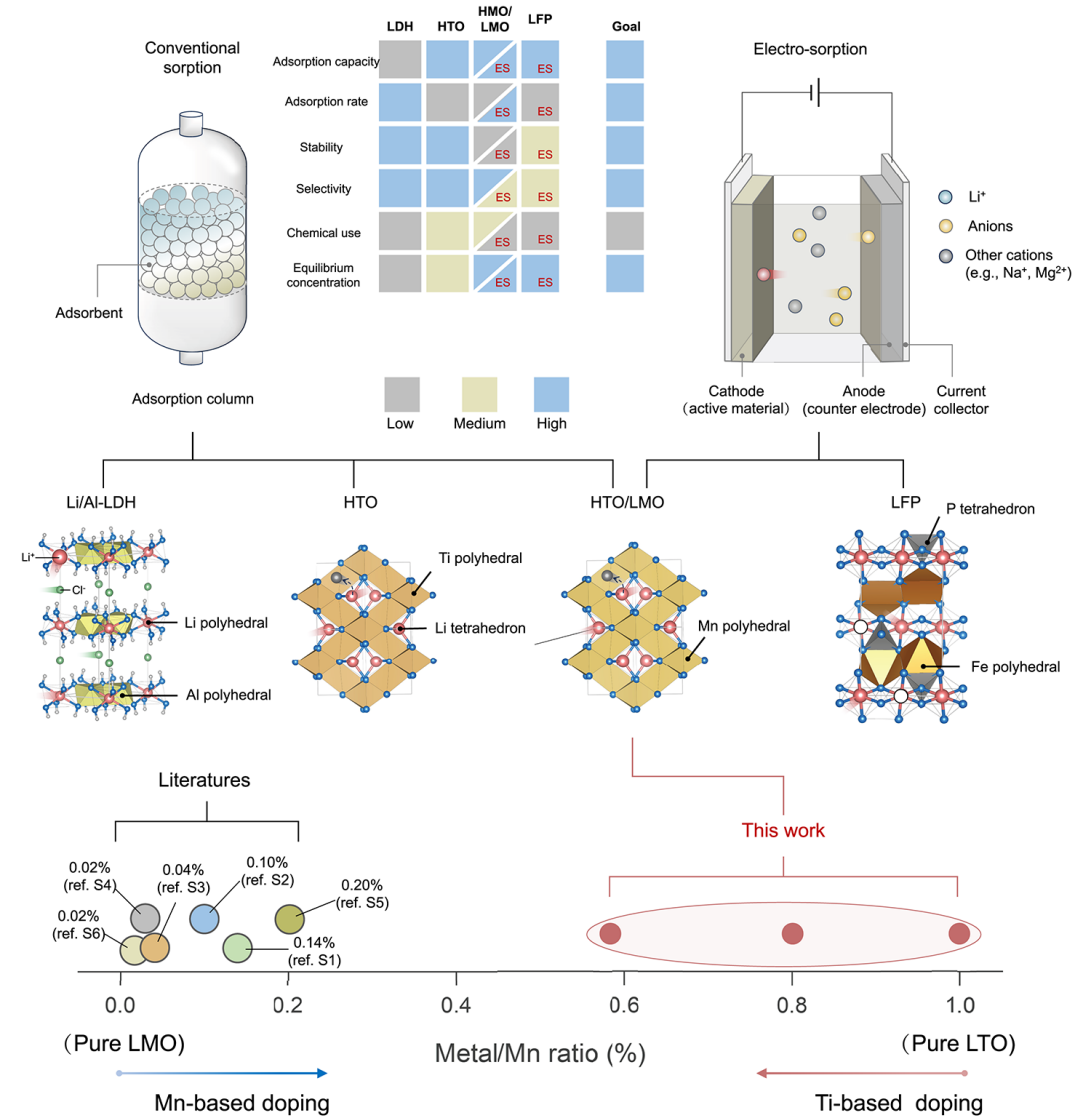
Limitations of existing adsorbent materials for DLE. Four major types of Li-selective materials are compared: lithium–aluminum layered double hydroxide (Li/Al-LDH), lithium titanate (LTO), lithium manganese oxide (LMO), and lithium iron phosphate (LFP). Li/Al-LDH and LTO are typically used as conventional ion-exchange adsorbents, whereas LFP functions as an electroactive sorbent in electrosorption-based DLE. LMO can serve both as a conventional adsorbent and as an electrode material. The heatmap summarizes the advantages and disadvantages of the four adsorbent materials across various performance metrics, including capacity, kinetics, stability, selectivity, and process-related constraints. No existing material demonstrates a uniformly high performance across all metrics. We hypothesize that Mn-doped LTO (namely, lithium manganese titanate (LTMO)) can overcome these limitations and serve as a high-performance electrode material for electrosorption.

Elemental substitution of spinel LMO has been widely explored, and Ti-substituted LMO is reported to alleviate part of the Mn^3+^-related distortion and improve cycling. Specifically, modifications of LMO with up to 20% Ti doping have been attempted (Figure 1 and Table S1).^12^ However, because the Ti-substituted LMO frameworks remain Mn-dominant, they still face similar challenges of LMO such as Jahn–Teller-driven structural changes, Mn dissolution in chloride-rich brines, and a practical redox window that partially overlaps the water splitting potential. While recent advances in spinel-based DLE have focused on compositional tuning and structural engineering, the fundamental principle of activating an electrochemically inert spinel host through site-selective doping remains underexplored.^13^ Instead of trying to improve the stability via Ti doping, here, we propose an alternative approach of imparting electrochemical activity to an intrinsically stable LTO framework via doping it with Mn. Specifically, we adopt an orthogonal design principle: using LTO as a near-zero strain, dissolution-resistant host while introducing a small fraction of Mn solely to provide redox activity within the aqueous window. We hypothesize that the Mn-doped LTO can enable fast, selective, and reversible Li uptake at neutral pH, rendering it an ideal candidate for electrochemical DLE.

In this work, we activate LTO via Mn doping to develop a stable and high-performance Li-selective electrode based on lithium titanium manganese oxide (LTMO). As both LTO and LMO belong to the spinel family, Mn can be incorporated into the LTO lattice without disrupting the overall crystal structure. We systematically evaluate the relationship between Mn doping levels and the electrode’s performance in electrosorption for Li extraction. We also investigated the structural transformations of LTMO to demonstrate how Mn substitution at specific crystal sites enhances the surface reaction kinetics and bulk ionic conductivity of the material. Density functional theory (DFT) simulations are employed to elucidate the relationship among the crystal structure, electronic properties, and performance of LTMO in electrochemical Li extraction. Finally, we perform electrosorption experiments using LTMO electrodes with real salt lake brines to demonstrate the electrodes’ high stability, fast Li uptake kinetics, and excellent selectivity. Notably, this work departs from conventional doping strategies that attempt to stabilize electroactive but less stable lattices (e.g., Ti-doped LMO). Unlike Ti-doped LMO, which only marginally delays structural decay, Mn-doped LTO represents a paradigm shift: converting an electrochemically inert but stable framework into a deliberately activated redox host that retains its structural stability.

## 2 Experiments and Calculation Methods

### 2.1 Adsorbent Synthesis

The precursor Li_1.81_H_0.19_Ti_2_O_5_·2H_2_O was synthesized by a hydrothermal method according to our previous work.^14^ Subsequently, Li_1_._33_Ti_x_Mn_y_O_4_ (denoted as LTMO) was obtained by calcining a mixture of C_4_H_14_MnO_8_ and the precursor at 600 °C for 3 h. LTMO particles were acid-treated with HCl to leach out lithium, thereby obtaining delithiated HTMO and concurrently activating its surface adsorption sites.

### 2.2 Electrode Preparation

Composite working electrodes were fabricated using electrospinning.^15^ A homogeneous dispersion was prepared by mixing 80 wt % HTMO, 10 wt % poly­(vinylidene fluoride) (PVDF, *M*_W_ = 10,00,000 g/mol), and 10 wt % carbon nanotubes (CNTs) in a solvent mixture of acetone and N,N-dimethylformamide (DMF) at a volume ratio of 2:1. The dispersion was electrospun at an applied voltage of 12 kV and a flow rate of 1 mL/h, with a tip-to-collector distance of 15 cm. The fibrous nanofiber mats were collected on a titanium plate (8 × 8 cm^2^) and dried to remove residual solvents. To enhance hydrophilicity, the films were coated with polydopamine (PDA) by immersing them in a PDA solution (10 mM, pH 8.5, in Tris buffer) for 6 h, followed by rinsing with deionized water. The counter electrodes were prepared using the same procedure but without any adsorbent particles and with CNTs as the sole active material, with a binder matrix composed of 10 wt % polyvinylidene fluoride (PVDF) and 90 wt % CNTs.

### 2.3 Material Characterization

X-ray diffraction (XRD) patterns were recorded by using a Rigaku SmartLab diffractometer (9 kW) with Cu Kα radiation (λ = 1.54 Å). The particle morphology and microstructure were characterized by scanning electron microscopy (SEM, JSM-6100LV, JEOL, Japan) and transmission electron microscopy (TEM, FEI Tecnai G2 F30). Elemental distribution was determined using elemental mapping with energy-dispersive X-ray spectroscopy (EDS), and the particle composition was determined by first dissolving the particles with hydrochloric acid (12 M) and then measuring the solution composition using inductively coupled plasma optical emission spectrometry (ICP-OES). Specific surface area measurements were performed using nitrogen adsorption/desorption isotherms (ASAP 2020, Meimaike). The optical band gap was evaluated by ultraviolet-Visible (UV–vis) diffuse reflectance spectroscopy (Shimadzu UV-3600, Japan). X-ray photoelectron spectroscopy (XPS, Thermo Scientific instrument) equipped with a monochromatic Al Kα source (1486.6 eV, 100 W) was employed to investigate surface chemical states and elemental valences before and after electrosorption.

### 2.4 Electrochemical Characterizations and Electrosorption Performance Test

Cyclic voltammetry (CV) was conducted on a CHI 660E electrochemical workstation (Chenhua, Shanghai, China) within a potential window of 0.3–1.1 V (vs saturated AgCl electrode). Electrochemical impedance spectroscopy (EIS) was used over the frequency range of 10^5^ to 10^–2^ Hz. The capacity and adsorption rate of different working electrodes were evaluated in a flow-through electrochemical cell. An adsorbent-loaded working electrode and CNT counter electrode with an effective area of 16 cm^2^ (4 × 4 cm^2^) were used. The feed solution (30 mL, pH 9.2) was circulated through the cell at a flow rate of 6 mL/min by using a peristaltic pump (BT100–1L, Longer Precision Pump Co., Ltd.). LiCl solutions of varying concentrations (40–333.17 mg/L) were used to obtain the isotherms. For measuring the adsorption rates, we used a Li solution with a concentration of 166.6 mg/L.

We also used real brine collected from Lagoco Salt Lake (Tibet) to test the performance of the best-performing HTMO electrode (H_1_._33_Ti_1_._17_Mn_0_._5_O_4_). The composition of the Lagoco Salt Lake brine is reported in Table S6 (Supporting Information). In measuring isotherms, adsorption rates, and electrosorption performance with real brine, the cell was operated with a constant voltage of 1.2 V applied using an electrochemical workstation (Chenhua, Shanghai, China).

### 2.5 Adsorption Isotherms and Kinetics

In general, the mass of lithium (Li) per mass of adsorbent (unit: mg/g) is denoted as *q*_*t*_ at any given time *t* and referred to as the adsorption amount, which can be quantified using the expression

$$q_{t}=\frac{\left(C_{0}-C_{t}\right)V}{m}$$

where *C*_0_ and *C*_*t*_ are the adsorbate concentration in the solution initially (*C*_0_) and at time *t* (*C*_*t*_), respectively, *V* is the solution volume, and *m* is the mass of the adsorbent. The adsorption capacity (*q*_*e*_) is defined as the adsorption amount when the system reaches equilibrium, which can be estimated using the expression

$$q_{e}=\frac{\left(C_{0}-C_{e}\right)V}{m}$$

where *C*_*e*_ is the adsorbate concentration in the solution when the adsorption process reaches equilibrium.

### 2.6 Computational Methods

First-principles calculations based on density functional theory (DFT) were performed by using the Vienna Ab initio Simulation Package (VASP). The interactions between ions and electrons were described by using the projector-augmented wave (PAW) method. The exchange–correlation functional was treated using the Perdew–Burke–Ernzerhof (PBE) formulation within the generalized gradient approximation (GGA). A plane-wave cutoff energy of 420 eV and a 3 × 3 × 1 Monkhorst–Pack k-point mesh were used. The convergence thresholds for energy and force were set to 10^–5^ eV and 0.02 eV/atom, respectively. Surface configurations of the Mn-substituted LTO were constructed by replacing Ti atoms with Mn on the (111) surface and applying a 15 Å vacuum layer to eliminate periodic interactions. The adsorption energy (*E*_ads_) of Li­(H_2_O)_4_^+^ on the HTMO-R surface was calculated by using the equation

$$E_{\mathrm{ads}}=E_{\mathrm{Li}(H_{2}O{)}_{4}^{+}/\mathrm{surface}}-E_{\mathrm{Li}(H_{2}O{)}_{4}^{+}}-E_{\mathrm{surface}}$$

where the energies of the adsorbed system, Li­(H_2_O)_4_^+^, and the clean surface are denoted as *E*_Li(H_2_O)_4_^+^/surface_, *E*_Li(H_2_O)_4_^+^_, and *E*_surface_, respectively.

## 3 Results and Discussion

### 3.1 Structurally Stable Mn–Ti Spinel Frameworks via Lattice Substitution

The performance of LTO-based spinel electrodes in electrochemical Li extraction is intrinsically limited by their ionic and electronic transport properties. To overcome these limitations and to improve Li^+^ diffusion kinetics, we employed a lattice site regulation strategy to partially substitute titanium (Ti) in the LTO crystal lattice with manganese (Mn) to modulate the crystal structure (Figure 2a). Crystalline Li_1_._33_Ti_x_Mn_y_O_4_ (LTMO) particles were prepared via a two-step method involving the hydrothermal synthesis of a LTO hydrate precursor (Li_1_._81_H_0_._19_Ti_2_O_5_·2H_2_O), followed by controlled calcination at 600 °C for 3 h with C_4_H_14_MnO_8_ to obtain phase-pure, spinel-structured LTMO via solid-state reaction. Acid leaching enabled the extraction of loosely bound Li^+^ ions from the spinel framework without compromising its crystallinity, producing delithiated hydrogen titanium manganese oxide (HTMO).

**Figure 2:**
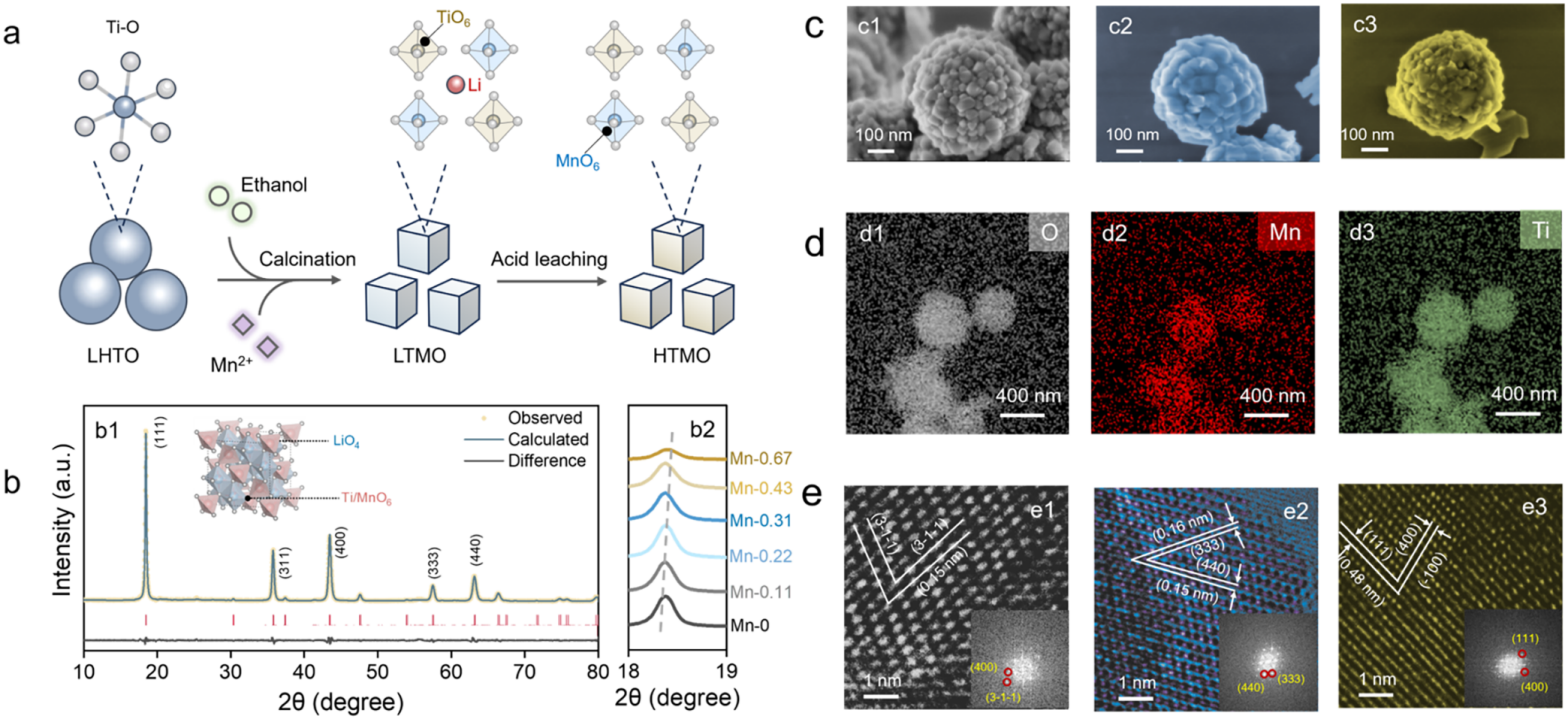
Synthesis, structure, and morphological characterization of Mn-substituted HTMO electrodes. (a) Schematic illustration of the synthesis process and structural evolution of Li_1_._33_Ti_x_Mn_y_O_4_-based materials. (b) Rietveld refinement of Li_1_._33_Ti_1_._17_Mn_0_._5_O_4_, showing good agreement between experimental and fitted patterns (b1), with the (111) peak displaying a rightward shift due to Mn substitution (b2). (c) Scanning electron microscopy (SEM) images of electrospun composite electrodes based on (c1) H_1_._33_Ti_1_._67_O_4_, (c2) H_1_._33_Ti_1_._37_Mn_0_._3_O_4_, and (c3) H_1_._33_Ti_1_._17_Mn_0_._5_O_4_. (d) Elemental mapping of H_1_._33_Ti_1_._17_Mn_0_._5_O_4_ via energy-dispersive X-ray spectroscopy (EDS), showing a homogeneous distribution of (d1) oxygen, (d2) manganese, and (d3) titanium throughout the material cross-section. (e) High-resolution transmission electron microscopy (HRTEM) images of (e1) Li_1_._33_Ti_1_._67_O_4_, (e2) Li_1_._33_Ti_1_._37_Mn_0_._3_O_4_, and (e3) Li_1_._33_Ti_1_._17_Mn_0_._5_O_4_, confirming the preservation of spinel crystal planes upon Mn substitution.

To verify the selective substitution of Ti by Mn and its effect on the bulk structure, we performed X-ray diffraction (XRD) analysis. A progressive rightward shift in the (111) peak position was observed as the Mn content increased (Figures 2b,S1), consistent with lattice contraction due to the smaller ionic radius of Mn^3+^/Mn^4+^ relative to Ti^4+^. Rietveld refinement confirmed the phase purity and the absence of secondary phases. Elemental compositions measured by energy-dispersive X-ray spectroscopy (EDS) were consistent with the target chemical formulas (e.g., Li_1_._33_Ti_1_._17_Mn_0_._5_O_4_, Table S2). The close agreement between nominal and measured Mn/Ti ratios confirms that Mn was successfully incorporated into the spinel lattice in a controlled manner rather than being introduced as a surface species. Importantly, the incorporation of Mn into TiO_6_ octahedra leads to the formation of MnO_6_ coordination environments (Figure 2a), which not only alters the average bond lengths and electronic structure but also introduces redox-active sites that play a pivotal role in subsequent Li^+^ intercalation dynamics (Table S3). Although the absolute changes in bond lengths are subtle (typically <1%), their systematic and directional trends, the progressive shortening of Li–O bonds and the elongation of Ti–O bonds with increasing Mn content, are highly indicative of local lattice distortion. Such coordinated adjustments, reliably captured by DFT calculations that are well-suited for predicting relative trends, can significantly modulate the local electric field and migration barriers for Li^+^ ions. These atomistic-level distortions provide a structural rationale for the dramatically enhanced Li^+^ diffusivity observed in electrochemical measurements, thereby bridging atomic-scale structural modulation with a macroscopic performance enhancement. Scanning electron microscopy (SEM) images reveal that both the delithiated LTO and LTMO particles are hierarchical aggregates of hundreds of nanometers, with primary crystal particles of tens of nanometers (Figure 2c). This hierarchical structure is likely favorable for Li^+^ adsorption kinetics due to its porosity and enhanced surface area as compared with monolithic crystal particles of hundreds of nanometers. Elemental mapping with EDS also reveals that Ti, Mn, and O are uniformly distributed throughout the HTMO aggregates (Figure 2d), suggesting that Mn is uniformly integrated within the spinel lattice rather than being surface-segregated. Moreover, high-resolution transmission electron microscopy (Figure 2e) reveals well-defined lattice fringes (0.48 nm spacing matching the (111) plane of spinel LTMO) and dominant (111) surface facets.

To assess the structural stability of the delithiated adsorbents, acid leaching experiments were also performed to remove Li from the LTMO particles. The near-complete removal of Li (>98%) and minimal dissolution of Ti (<0.2%) and Mn (<0.5%) from the optimized LTMO adsorbents suggest that the spinel structure remains stable after Li/proton exchange (Figure S2). Among the five compositions evaluated, the degrees of Ti and Mn dissolution were minimized when the Mn/Ti molar ratio was 0.43 (i.e., Li_1_._33_Ti_1_._17_Mn_0_._5_O_4_). This composition was thus selected for more comprehensive characterization and performance evaluation, along with Li_1_._33_Ti_1_._37_Mn_0_._3_O_4_ with a Mn/Ti molar ratio of 0.22, primarily for comparison. This optimal Mn/Ti ratio (≈0.43) achieves the best balance between Mn-induced redox activity and the structural stability of the Ti-rich framework. A higher Mn content would shift the material toward conventional Mn-dominant spinels, compromising the stability that is central to our design strategy of activating an inert host.

### 3.2 Mn Doping Enhances Electrochemical Lithium Adsorption Capacity and Kinetics

To evaluate the performance of LTMO-based electrodes for selective electrosorption of Li, composite electrodes were fabricated via electrospinning using HTMO as the electroactive selective adsorbent, polyvinylidene fluoride (PVDF) as the binder, and carbon nanotubes (CNTs) to enhance the electrical conductivity (Figure 3a and Methods).^16^ The electrospun fibrous architecture was employed to construct an open, three-dimensional (3D) electrode scaffold. This design facilitates electrolyte infiltration and ion transport, provides a continuous conductive network via the embedded CNTs, and helps mitigate particle agglomeration, collectively contributing to enhanced electrochemical kinetics.^17^ The impact of site regulation on electrochemical behavior was first assessed by using cyclic voltammetry (CV) with the composite film electrodes. With increasing Mn content in the LTMO particles, peak current intensities increased and redox features sharpened, reflecting enhanced Li uptake and reduced polarization (Figure 3b).^18^ Scan rate-dependent cyclic voltammetry (CV) analysis revealed a linear relationship between the peak current (*i*_*p*_) and the square root of the scan rate (*v* ^1/2^) (Figure S3), which is indicative of a diffusion-controlled intercalation process rather than surface-limited pseudocapacitive behavior. The appearance of distinct redox peaks in Mn-substituted samples further confirms the participation of reversible Mn^3+^/Mn^4+^ redox couples during the Li^+^ ion uptake. According to electrochemical impedance spectroscopy (EIS), both the charge transfer and ion diffusion resistances of HTO are substantially reduced by Mn doping. Specifically, compared to the undoped HTO electrodes, the optimized HTMO (H_1_._33_Ti_1_._17_Mn_0_._5_O_4_) exhibits a 57% reduction in charge transfer resistance (2.96 Ω vs 5.26 Ω) and a 75% decrease in the slope of the Warburg region (2.90 vs 11.6) (Figure 3c, Table S4). These findings suggest that Mn incorporation facilitates redox activity and reduces ion transport barriers, contributing to the superior electrochemical performance observed in HTMO. For a quantitative benchmark, the optimized HTMO electrode exhibits a Li^+^ diffusion coefficient (2.15 × 10^–11^ cm^2^/s) comparable to that of conventional LiMn_2_O_4_ (3.97 × 10^–11^ cm^2^/s, Table S4), while its charge transfer resistance (2.96 Ω) is moderately higher than that of LiMn_2_O_4_ (1.62 Ω). Crucially, HTMO achieves this kinetic performance with drastically reduced Mn dissolution (0.73 wt % vs 15.2 wt % over ten cycles), demonstrating an optimal balance for durable DLE operation.

**Figure 3:**
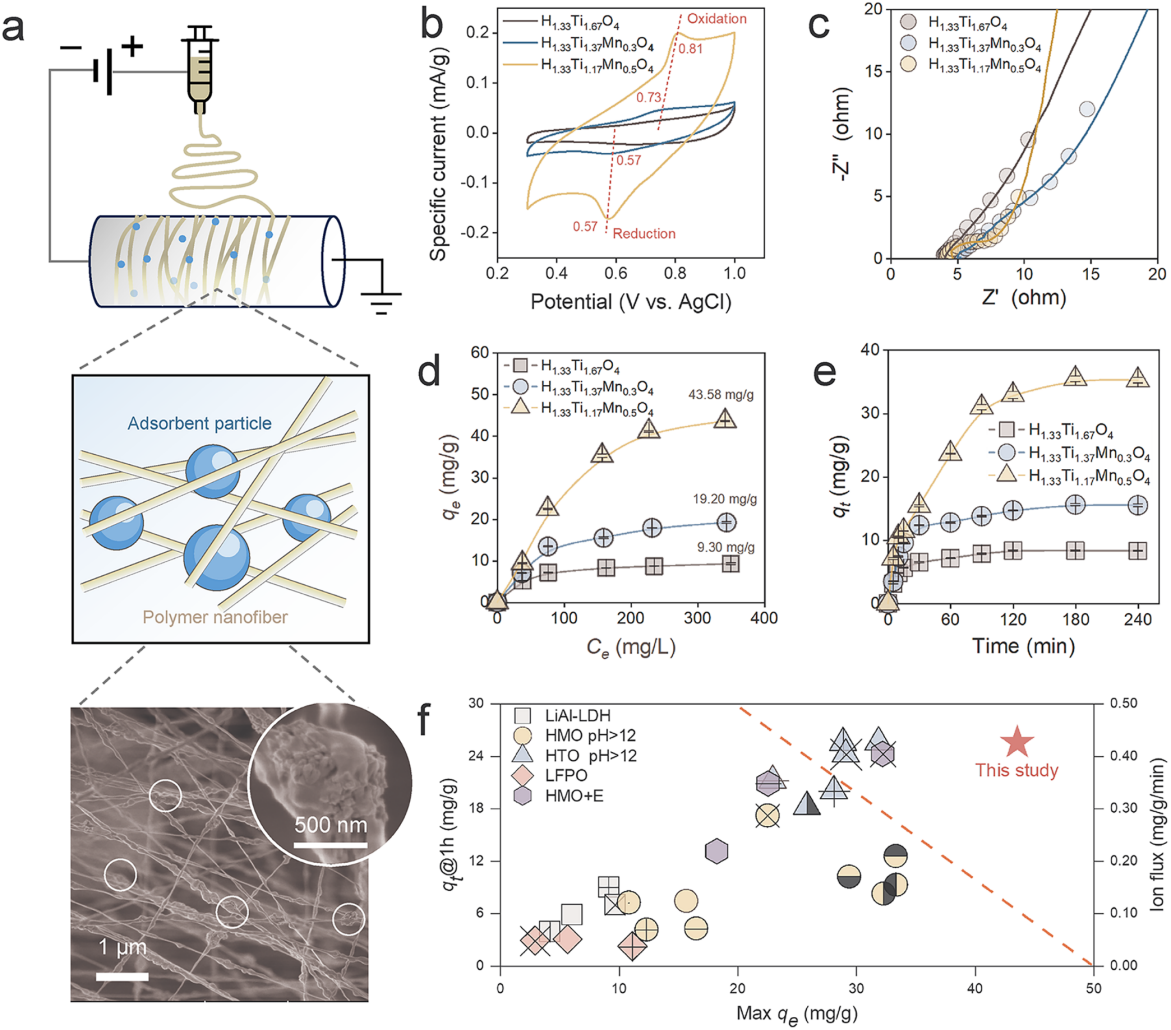
Fabrication and performance evaluation of Mn-substituted HTMO film electrodes. (a) Formation of film electrodes by electrospinning of HTO or HTMO adsorbent particles, carbon nanotubes (CNTs), and poly­(vinylidene fluoride) (PVDF) to form a composite fibrous structure with adsorbent particles embedded as shown in the scanning electron microscopy image. (b) Cyclic voltammograms for the three electrode materials. The Li_1_._33_Ti_1_._17_Mn_0_._5_O_4_ electrode has considerably higher electrochemical activity than that of the undoped Li_1_._33_Ti_1_._67_O_4_ electrode. (c) Nyquist plots from electrochemical impedance spectroscopy for the three electrode materials. (d) Adsorption isotherms for the three electrode materials with an applied voltage of 1.2 V (counter electrode: C). (e) Adsorption rates for the three electrode materials with an applied voltage of 1.2 V in a LiCl solution (166.67 mg/L). (f) Maximum equilibrium adsorption capacity vs adsorption rate (first hour) for HTMO vs adsorbents/electrodes reported in the literature. HTMO was tested in real Lagoco salt lake brine, while most literature values were obtained from synthetic solutions. Testing conditions are summarized in Table S6.

To evaluate Li^+^ uptake under relevant operating conditions, batch adsorption experiments were conducted at a constant potential of 1.2 V to assess both isotherms and adsorption rates. The delithiated H_1_._33_Ti_1_._17_Mn_0_._5_O_4_ electrode demonstrates a remarkably high adsorption capacity of 43.58 mg/g (Figure 3d), surpassing Li adsorbent materials (whether electroactive or not) under comparable conditions.^19^ With an applied voltage of 1.2 V in a 166.6 mg/L LiCl solution, we evaluated the adsorption rates using undoped HTO and HTMO electrodes with two levels of Mn doping (Figure 3e). In the relevant time scale for electrochemical Li interaction (>60 min for the charging step), the H_1_._33_Ti_1_._17_Mn_0_._5_O_4_ electrode achieved a much higher adsorption rate because the HTO and the nonoptimized HTMO electrode (H_1_._33_Ti_1_._37_Mn_0_._3_O_4_) already approached saturation with much lower capacities. The first-hour adsorption rate of the optimized H_1_._33_Ti_1_._17_Mn_0_._5_O_4_ electrode (1.2 V) is 4- to 6-fold that of conventional LiAl-LDH adsorbents and is only matched by HTO under highly alkaline conditions (pH > 12) or electrosorption with hydrogen manganese oxide (HMO) electrodes (Figure 3f and Tables S6,S7). Besides crystal structure modulation induced by Mn doping that reduces ion diffusion and charge transfer resistances, the adsorption kinetics may further be enhanced by the larger specific surface area of the HTMO particles (52.64 vs 40.08 m^2^/g for HTO, Figure S5) possibly due to the smaller size of primary particles in the hierarchical aggregates. This textural advantage is directly validated by electrochemical impedance spectroscopy, which shows a concomitant increase in the Li^+^ diffusion coefficient by 2–3 orders of magnitude (Table S4), quantitatively confirming that the Mn-induced porosity facilitates rapid ion transport.

### 3.3 Structural Modulation by Mn Facilitates Lithium Storage Transport in Spinel Electrodes

When used as a conventional adsorbent (without applied voltage), the Li adsorption capacity of undoped HTO (in a film with CNT/PVDF) is only 5.02  mg/g. With an applied voltage (1.2 V), the Li adsorption capacity of HTO increases to ∼8.31 mg/g (Figures 4a andS5). The Mn-doped HTMO particles have similar Li adsorption capacities when used as a conventional adsorbent (4.98 mg/g for H_1_._33_Ti_1_._37_Mn_0_._3_O_4_ and 6.15 mg/g for H_1_._33_Ti_1_._17_Mn_0_._5_O_4_), but their Li adsorption capacities as electrodes (at 1.2 V) are dramatically increased (15.53 mg/g for H_1_._33_Ti_1_._37_Mn_0_._3_O_4_ and 35.25  mg/g for H_1_._33_Ti_1_._17_Mn_0_._5_O_4_). The substantial increase of the adsorption capacity for the HTMO electrodes, compared either to the same materials when used as a conventional adsorbent or to HTO as an electrode material, highlights the important role of Mn^4+^/Mn^3+^ valence modulation in enhancing the Li^+^ storage capacity of the spinel crystal structure. For the optimized HTMO electrode (H_1_._33_Ti_1_._17_Mn_0_._5_O_4_), activation by Mn doping unlocks more than 85% of the capacity inaccessible to the undoped HTO crystal structure.

**Figure 4:**
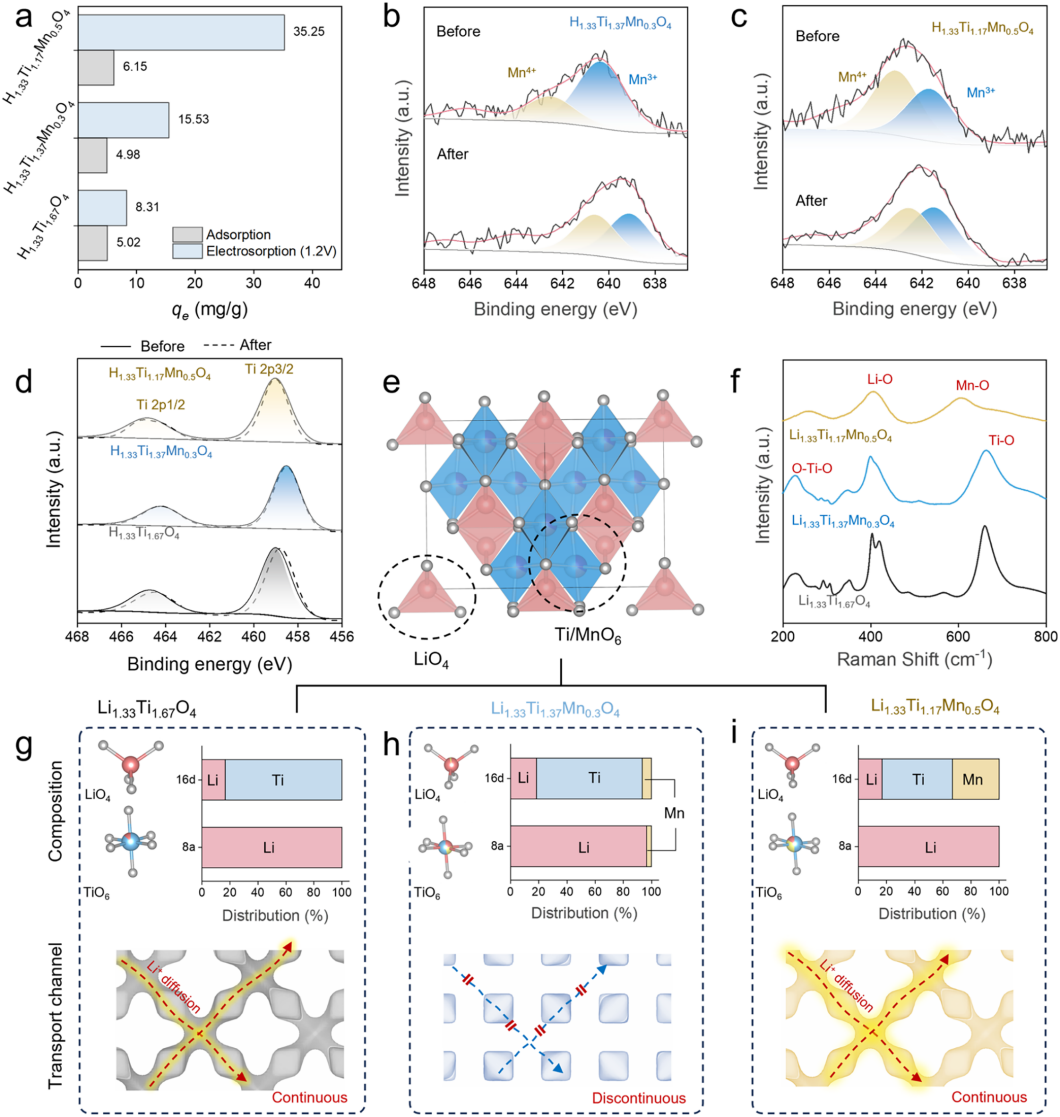
Structural and electronic evolution of HTMO and its impact on Li^+^ diffusion pathways. (a) Electrochemical and nonelectrochemical lithium adsorption capacities of HTMO-based materials with varying Mn content. (b, c) X-ray photoelectron spectroscopy (XPS) results of Mn 2p core-level spectra. Mn 2p spectra reveal a mixed-valence Mn^3+^/Mn^4+^ state, with increased Mn^3+^ content in more heavily doped HTMO, supporting its role as a redox-active center. (d) Ti 2p spectra remain unchanged with doping, confirming that Ti stays in the Ti^4+^ state and is electrochemically inactive. (e) Schematic of the spinel lattice framework showing Li^+^, Ti^4+^, and Mn^4+^ occupancy in tetrahedral (8a) and octahedral (16d) sites, along with representative Li^+^ diffusion channels. (f) Raman spectra showing the emergence of Mn–O vibrational modes and the distortion of Ti–O and Li–O peaks with increasing Mn content, indicating changes in local coordination environments. Structural model and cation site occupancy for (g) undoped hydrogen titanate (Li_1_._33_Ti_1_._67_O_4_), (h) Li_1_._33_Ti_1_._37_Mn_0_._3_O_4_, and (i) Li_1_._33_Ti_1_._17_Mn_0_._5_O_4_. The presence of Mn in the 8a site for lightly doped HTMO (Li_1_._33_Ti_1_._37_Mn_0_._3_O_4_) disrupts the Li^+^ transport network that is continuous in undoped HTO and the more heavily doped HTMO (Li_1_._33_Ti_1_._17_Mn_0_._5_O_4_) due to the absence of Mn in the 8a sites.

To gain more mechanistic insights regarding how Mn doping influences the electrode’s electrochemical behavior, X-ray photoelectron spectroscopy (XPS) was used to probe the surface composition and local bonding environment before and after Li^+^ intercalation. The XPS spectra confirm the presence of Ti, Mn, and O as the dominant elements, with no detectable impurities or elemental loss after cycling (Figure S6). High-resolution O 1s spectra reveal an increased contribution from lattice oxygen (O–Ti/Mn) and a corresponding decrease in adsorbed water and surface hydroxyl species upon Li^+^ uptake (Figure S7), indicating a transition toward a more ordered coordination environment dominated by lattice oxygen. Valence state analysis based on XPS further shows that Mn^4+^ is partially reduced to Mn^3+^ during Li^+^ intercalation (Figure 4b,c), while Ti remains electrochemically inactive (Figure 4d), identifying Mn as the primary redox-active center. The simultaneous modulation of Mn valence and oxygen coordination supports a redox-coupled Li^+^ intercalation mechanism distinct from the classical ion-exchange process in undoped HTO.^20^ This reversible redox process contributes to enhanced structural stability during repeated Li^+^ intercalation and deintercalation. Notably, the reversible Mn^3+^/Mn^4+^ redox occurs without triggering significant Jahn–Teller lattice distortion, which is a common cause of degradation in Mn-rich spinels (e.g., LMO). This stability is attributed to the Ti^4+^-dominated framework: the JT-inactive Ti^4+^ ions dilute the Mn^3+^ centers, disrupt the long-range Mn–O–Mn connectivity required for cooperative distortion, and thus stabilize the local octahedral environment. This mechanism is consistent with the high structural integrity and minimal Mn dissolution observed during cycling.

In principle, Mn dopants can occupy both the tetrahedral 8a sites, typically coordinated as LiO_4_, and the octahedral 16d sites, corresponding to TiO_6_ (Figure 4e). Given that the 8a sites are integral to Li^+^ diffusion pathways, their partial occupation by Mn is expected to impede ion mobility. To understand the impacts of Mn incorporation on crystallographic site occupancy and long-range Li^+^ ion transport, Raman spectroscopy was used to probe changes in short-range coordination environments. The spectra reveal a gradual shift in vibrational features with increasing Mn content, including the emergence and intensification of Mn–O bands in the 500–650 cm^–1^ region and concurrent changes in the Li–O and Ti–O vibrational modes (Figure 4f). These spectral evolutions suggest that higher Mn concentrations favor the redistribution of Mn from tetrahedral 8a to octahedral 16d sites, accompanied by distortions in the Li–O and Ti–O bonding environments.

To further elucidate the impact of Mn concentration on Mn site preference and its consequent impact on long-range Li^+^ transport, we employed bond-valence sum difference mapping (BVSDM) to simulate Li^+^ migration pathways in Mn-substituted spinel lattices (Figures 4g–i andS8). For Li_1.33_Ti_1.67_O_4_, the 8a sites are exclusively occupied by Li^+^ ions seated in a tetrahedral (LiO_4_) coordination (Figure 4g), forming a three-dimensional network that facilitates fast Li^+^ migration. In H_1_._33_Ti_1_._37_Mn_0_._3_O_4_, however, BVSDM simulations suggest that partial occupation of the 8a sites by Mn disrupts Li^+^ transport pathways (Figure 4h). At an even higher Mn concentration (H_1_._33_Ti_1_._17_Mn_0_._5_O_4_), a fully connected and low-energy network for Li^+^ transport is restored as 8a sites become predominantly occupied by Li again, while Mn–Ti co-occupancy stabilizes the 16d octahedral sites, creating a coherent framework (Figure 4i). These BVSDM simulation results are consistent with Mn-dependent spectral evolution observed in Raman spectroscopy.

Taken together, these results reveal a cascade of effects initiated by Mn substitution—from selective site occupation and local coordination changes to redox-enabled charge compensation and the reopening of Li^+^ transport channels. These structural and electronic modulations work synergistically across length scales to enhance both the Li^+^ storage capacity and transport kinetics. Such a multifaceted regulation of the structure and function underpins the superior electrochemical performance observed in Mn-doped HTMO electrodes and offers a rational design framework for developing next-generation materials for electrosorption-driven lithium extraction.

### 3.4 Electronic Mechanism behind Enhanced Lithium Adsorption in Mn-Doped Spinels

In addition to modulating lattice site occupancy and transport pathways, Mn doping may fundamentally alter the electronic landscape of the host material, thereby contributing to the enhanced redox activity and Li^+^ adsorption performance observed in the electrochemical measurements. Since Li^+^ intercalation involves coupled ion–electron transfer and is highly sensitive to the host’s electronic structure, understanding these effects is critical to rational material design. To investigate these electronic effects, we performed a combination of spectroscopic analysis and density functional theory (DFT) calculations.

A progressive narrowing of the optical band gap was identified by ultraviolet–visible (UV–vis) absorption spectroscopy, as evidenced by a red shift in the absorption edge from 420 nm in undoped HTO to 480 nm in Mn-doped HTMO (Figures 5a andS9). Tauc plot analysis confirms a corresponding decrease in the band gap from 2.86 eV (Li_1.33_Ti_1.67_O_4_) to 1.27 eV (Li_1.33_Ti_1.17_Mn_0.5_O_4_), which suggests that Mn substitution promotes electron delocalization and thereby facilitates charge carrier transport within the spinel structure.^21^

**Figure 5:**
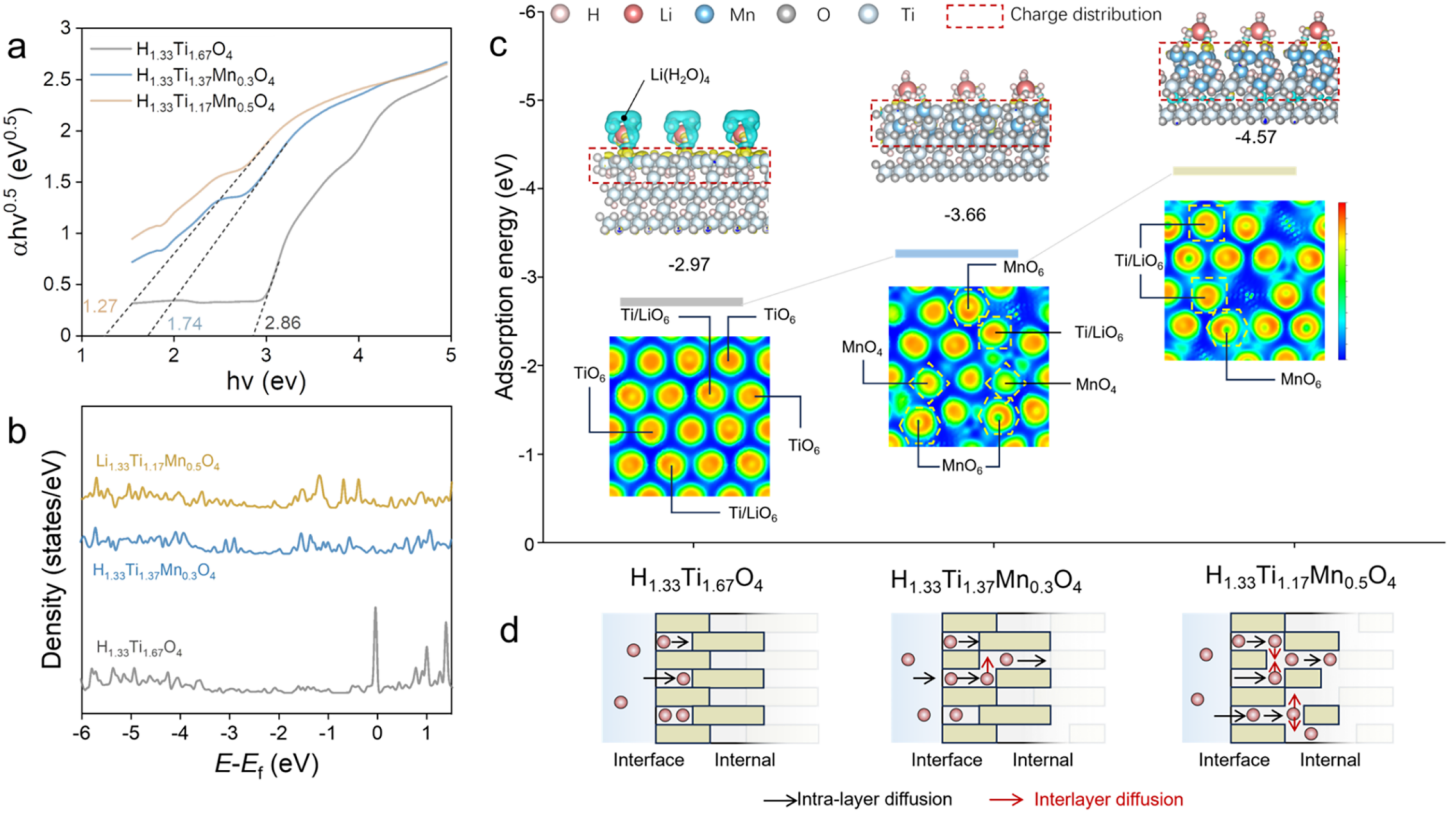
Mn-induced modulation of the electronic structure and Li^+^ interfacial behavior. (a) UV–vis absorption spectra show progressive band gap narrowing with increasing Mn content, indicating enhanced electronic delocalization. (b) DFT-calculated density of states (DOS) illustrates the emergence of additional electronic states near the Fermi level, consistent with improved charge transport properties. (c) Adsorption energy calculations and charge density maps of solvated Li^+^ reveal stronger interfacial Li^+^ binding and localized electron accumulation in Mn-rich systems. (d) Schematic illustration of proposed Li^+^ migration pathways, demonstrating how Mn-driven changes in the lattice structure and electronic environment facilitate more efficient ion transport.

DFT-calculated partial density of states (PDOS) revealed that Mn incorporation introduces additional d-states near the Fermi level, broadening the conduction band and increasing the density of accessible electronic states (Figure 5b).^22^ This electronic restructuring is expected to improve the intrinsic conductivity and support more efficient redox reactions during electrochemical cycling. Besides the global changes in the band structure, charge density difference mapping also provides insights into the local bonding interactions. In Mn-substituted structures, we observe electron accumulation at Mn–O–Ti linkages, especially in Mn-rich compositions, indicating localized charge redistribution. These results suggest that the Mn dopant acts as both a charge modulator and an internal electron reservoir during Li^+^ intercalation, facilitating dynamic redox stabilization.

To evaluate how electronic reconfiguration affects the Li interaction at the interface, we calculated the adsorption energies of solvated lithium ions (Li­(H_2_O)_4_^+^) on various surfaces. The Mn-rich HTMO electrode exhibits the strongest binding affinity (−4.57 eV), compared to the less doped HTMO electrode (−3.66 eV) and the undoped HTO electrode (−2.97 eV) (Figure 5c). This favorable adsorption energy, coupled with localized charge accumulation around surface oxygen atoms, reflects an improved ability of the Mn-substituted surface to attract and stabilize Li^+^ during uptake.^23^ These findings indicate that Mn substitution enables site-specific tuning of both the bulk electronic structure and interfacial charge behavior. By narrowing the band gap, enriching redox-active electronic states, and strengthening surface Li^+^ binding, Mn serves as an electronic regulator that enhances both conductivity and interfacial reactivity to enable fast Li^+^ intercalation from the solution into the lattice (Figure 5d).

### 3.5 Selective and Stable Lithium Extraction from Real Brine Using Mn-Doped Spinel Electrodes

Besides demonstrating higher capacity and faster kinetics in controlled electrochemical tests (Figure 3c–h), the Mn-doped HTMO electrodes also exhibit exceptional performance in electrosorption-based DLE from chemically complex natural brine with high concentrations of competing Na^+^ and Mg^2+^ ions (Figure 6). We evaluated the performance of the optimized HTMO electrode (H_1_._33_Ti_1_._17_Mn_0_._5_O_4_) in a DLE treatment train based on electrosorption using a brine sourced directly from Lagoco Salt Lake in Tibet (see Table S8 for composition), a representative Li-rich salt lake characterized by high salinity and excessive competing cations. The DLE treatment train consists of four stages (Figure 6a): (i) prefiltration to remove suspended solids and organic matter for preventing electrode fouling; (ii) electrosorption–desorption using Mn-doped HTMO to enrich Li^+^ while removing the majority of competing Na^+^ and Mg^2+^; (iii) reverse osmosis (RO) and evaporation to concentrate the Li^+^ eluate generated in step (ii); and (iv) final crystallization to recover lithium as Li_2_CO_3_. The electrosorption process was operated in adsorption–desorption cycles (Figure 6b), involving Li^+^ intercalation under an applied voltage (1.2 V), followed by polarity reversal to release adsorbed Li^+^. A rinsing step was introduced between the adsorption half-cycle and desorption half-cycle to remove impurity ions (e.g., Na^+^, K^+^) trapped in the macropores, thereby reducing eluate contamination.

**Figure 6:**
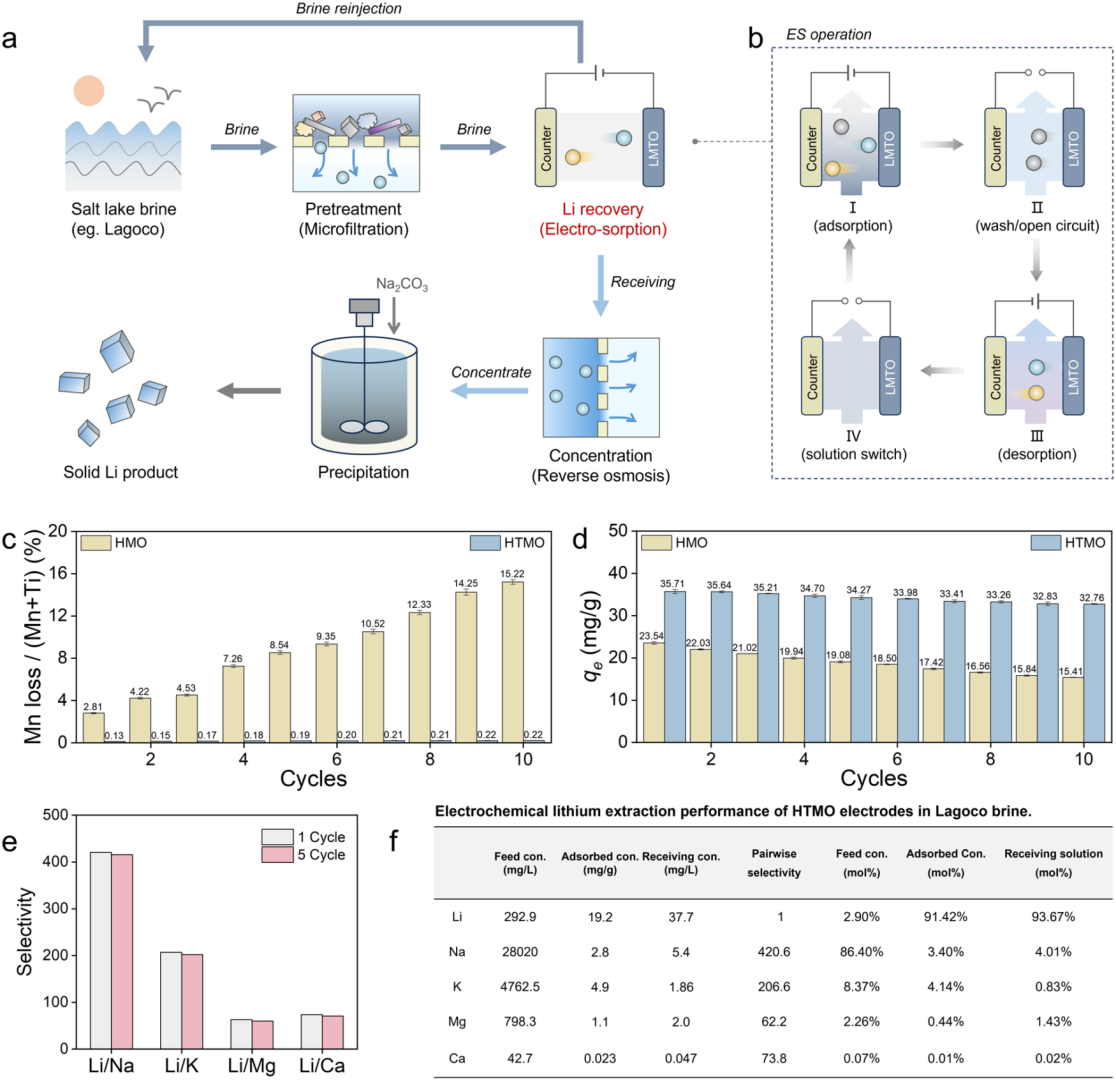
Selectivity and stability of Mn-doped HTMO electrodes in electrosorption-based Li extraction from real salt lake brine. (a) Schematic of an integrated DLE system using Mn-activated titanate (HTMO) electrodes as the selective Li^+^ capture components. The system was evaluated using high-salinity brine sampled from Lagoco Salt Lake, which contains elevated concentrations of Na^+^, K^+^, and Mg^2+^ as competing ions. (b) Electrosorption–desorption cycling protocol involving periodic polarity reversal and intermediate rinsing steps to maintain surface activity and minimize contamination across cycles. (c) Loss of Mn (vs Mn in the particles) from dissolution for HMO and HTMO electrodes during cycle processes. (d) Equilibrium Li^+^ adsorption capacity (left *y*-axis) and capacity loss (right *y*-axis) over five cycles for HTMO and HMO electrode. (e) Li^+^/M selectivity (M = Na^+^, K^+^, Mg^2+^, Ca^2+^) averaged across five electrosorption–desorption cycles measured using the Lagoco brine. (f) Feed concentrations, adsorbate concentrations (per mass of adsorbent), and receiving solution concentrations for the major cations in mg/L and mole fraction.

In electrosorption with the Lagoco brine, the Mn-doped HTMO electrodes exhibited a stable charging–discharge behavior over multiple cycles (Figure 6c,d). By integrating HMO’s electroactivity with HTO’s structural stability, the Mn-doped HTMO electrodes effectively mitigate the electrochemical instability. To quantify Mn loss, we measured the Mn concentration in the eluate using inductively coupled plasma mass spectrometry (ICP-MS). Over ten cycles of charging/discharge (∼120 h of operation), Mn loss from the HTMO electrode was only 0.73 wt % of the total Mn content in the electrode, dramatically lower than the more than 15.2 wt % loss observed for undoped HMO (Figure 6c). When normalized to the total 16d site cations rather than to Mn alone, the loss in HTMO corresponds to only 0.22% of 16d site cations, compared with 15.2% for HMO. Consequently, the HTMO electrode exhibited only a minor decline in equilibrium adsorption capacity, recording an ∼8% capacity loss after 10 cycles, which is in stark contrast to the staggering 35% loss in capacity for HMO (Figure 6d). The high electrochemical stability of the HTMO structure underpins the consistent capacity retention over multiple electrosorption–desorption cycles.^8,24^ This is further corroborated by ex situ XRD showing reversible lattice preservation (Figure S10) and SEM confirming the integrity of the fibrous network after cycling (Figure S11), with no signs of fouling or blockage, suggesting effective mitigation of particulate accumulation by the combined prefiltration and open electrode architecture. The 10-cycle test, corresponding to >120 h of continuous operation in real brine and a cumulative Li^+^ uptake of 49.28 mmol/g, demonstrates robust performance under extended testing conditions. This experimental depth exceeds the scope of the initial assessment commonly reported for novel electrode materials in direct lithium extraction, highlighting its promising durability for practical application.

In addition to enhancing capacity, kinetics, and electrochemical stability, HTMO electrodes also exhibit high selectivity toward Li^+^, yielding high separation factors (α) over competing ions in electrosorption tests using Lagoco Salt Lake brine with a complex composition. The material exhibits high separation factors for Li^+^ over competing ions in authentic Lagoco Salt Lake brine—α_Li/Na_ = 420.6, α_Li/K_ = 202.6, α_Li/Mg_ = 62.2, and α_Li/C*a*_ *=* 73.8—confirming excellent Li^+^ selectivity in a multi-ion environment. These values remained consistently high over five electrosorption/desorption cycles (Figure 6e), indicating stable selectivity under repeated cycling. The electrosorption/desorption process based on HMTO electrodes produced an eluate containing ∼ 94% Li^+^ (mole fraction) from a brine with an initial Li^+^ content of only 2.9% (Figure 6f), enabling efficient downstream processing (e.g., carbonate precipitation) in the DLE treatment train.

## 4 Implications

This study demonstrates that an electrochemically inert yet structurally robust spinel host can be transformed into a highly active and durable electrode through deliberate, site-selective redox activation. By introducing Mn into the octahedral (16d) sites of the lattice, the material achieves enhanced Li^+^ adsorption energetics, electronic conductivity, and reversible redox activity without sacrificing the structural integrity. This inert-to-active transformation resolves the classic activity–stability trade-off, establishing a broadly applicable design framework for coupling redox functionality and ion transport in aqueous electrochemical systems.

The Mn-activated lithium titanate electrode enables acid-free, energy-efficient lithium extraction from complex natural and industrial brines, including geothermal fluids, oilfield produced water, and battery recycling leachates, while eliminating corrosive reagents and minimizing secondary waste generation. By bridging structural stability with redox reactivity, this material design provides a scalable and environmentally benign pathway for lithium recovery from low-grade or high-salinity resources. Furthermore, the material’s composition is compatible with scalable synthesis routes (e.g., sol–gel, solid-state), supporting its potential transition beyond the laboratory-scale proof of concept demonstrated herein.

Beyond lithium, the principle of site-selective redox activation provides mechanistic insights into how targeted lattice modifications tune ion–electron coupling and interfacial energetics. This concept can guide the development of next-generation electrodes, ion-selective membranes, and electrochemical separation systems integrated with renewable energy, supporting the circular and low-carbon recovery of critical minerals central to the clean energy transition.

## Supplementary Material



## Notes

Detailed step-by-step calculation methodology for the Li^+^/M^+^ separation factors; structural evolution characterization via XRD patterns; chemical stability and acid leaching results for Li, Ti, and Mn, electrochemical characterization including scan rate-dependent cyclic voltammetry and diffusion kinetic analysis; BET surface area and BJH pore size distribution analysis, comparison of adsorption capacities under different voltage configurations; surface chemical state analysis via survey and high-resolution O 1s XPS spectra, bond valence-based Li^+^ migration pathway and percolation network simulations; optical band gap modulation study via UV–vis diffuse reflectance spectra, structural, chemical, and morphological stability of electrodes after cycling in real brine verified by ex situ XRD, Mn 2p XPS, and SEM analysis; and EDS elemental composition, local bond lengths, EIS kinetic parameters; and comprehensive performance comparisons with the literature (PDF)

## References

[1] Han Y., Xie W., Hill G. T., Smeets P., Hu X., Yan G., Zou S., Liu J., Wu R., Shi F. (2024). Uncovering the predictive pathways of lithium and sodium
interchange in layered oxides. Nat. Mater..

[2] Liu C., Li Y., Lin D., Hsu P.-C., Liu B., Yan G., Wu T., Cui Y., Chu S. (2020). Lithium Extraction from Seawater
through Pulsed Electrochemical Intercalation. Joule.

[3] DuChanois R. M., Cooper N. J., Lee B., Patel S. K., Mazurowski L., Graedel T. E., Elimelech M. (2023). Prospects
of metal recovery from
wastewater and brine. Nat. Water.

[4] Hill G. T., Shi F., Zhou H., Han Y., Liu C. (2021). Layer spacing gradient (NaLi)_1–*x*
_CoO_2_ for electrochemical Li extraction. Matter.

[5] Zhang L., Zhang T., Zhao Y., Dong G., Lv S., Ma S., Song S., Quintana M. (2024). Doping engineering of lithium-aluminum
layered double hydroxides for high-efficiency lithium extraction from
salt lake brines. Nano Res..

[6] Xu L., Zhao B., Zhang X., Liu W., Rau D., Wu D., Zhang W., Liu C., Liu Z., Lin S. (2025). Membrane and
electrochemical separations for direct lithium extraction. Nat. Chem. Eng..

[7] Yan G., Kim G., Yuan R., Hoenig E., Shi F., Chen W., Han Y., Chen Q., Zuo J. M., Chen W., Liu C. (2022). The role of
solid solutions in iron phosphate-based electrodes for selective electrochemical
lithium extraction. Nat. Commun..

[8] Li Z., Chen I.-C., Cao L., Liu X., Huang K.-W., Lai Z. (2024). Lithium extraction from brine through
a decoupled and membrane-free
electrochemical cell design. Science.

[9] Chen X., Wu C., Lv Y., Zhang C., Zhang X., Nie L., Zhang Y., Zhao L., Huang C., Liu W. (2022). Self-driven
lithium extraction by directional liquid transport nonwoven. Matter.

[10] Zhao X., Yang S., Song X., Wang Y., Zhang H., Li M., Wang Y. (2024). Enhanced Lithium Extraction
from Brines: Prelithiation
Effect of FePO_4_ with Size and Morphology Control. Adv. Sci..

[11] Kim H., Hong J., Park K. Y., Kim H., Kim S. W., Kang K. (2014). Aqueous rechargeable Li and Na ion
batteries. Chem. Rev..

[12] Fang D., Liu F., Zhang P., Zhang X., Li K. (2025). Leveraging LaMnO_3_ coated
and La-doped LiMn_2_O_4_ for enhanced electrochemical
lithium extraction stability. Desalination.

[13] Zhao X., Zheng L., Hou Y., Wang Y., Zhu L. (2022). Pulsed electric field controlled
lithium extraction process by LMO/MXene composite electrode from brines. Chem. Eng. J..

[14] Zhao B., Qian Z., Qiao Y., Li J., Wu Z., Liu Z. (2023). The Li­(H_2_O)_n_ dehydration behavior
influences
the Li^+^ ion adsorption on H_4_Ti_5_O_12_ with different facets exposed. Chem.
Eng. J..

[15] Su R., Li L., Kang J., Ma X., Chen D., Fan X., Yu Y. (2022). AgNPs-thiols modified PVDF electrospun nanofiber membrane with a
highly rough and pH-responsive surface for controllable oil/ water
separation. J. Environ. Chem. Eng..

[16] Ji D., Lin Y., Guo X., Ramasubramanian B., Wang R., Radacsi N., Jose R., Qin X., Ramakrishna S. (2024). Electrospinning
of nanofibres. Nat. Rev. Method Primers.

[17] Zhao B., Wei W., Qiao Y., Sheng X., Xu J., Qian Z., Lv B., Liu Z. (2025). Coordination Environment Engineering of Titanium Spinel
for Enhanced Lithium Recovery. Nano Lett..

[18] Liu X., Xu X., Xuan X., Xia W., Feng G., Zhang S., Wu Z. G., Zhong B., Guo X., Xie K., Yamauchi Y. (2023). Unlocking Enhanced Capacitive Deionization of NaTi_2_(PO_4_)_3_)/Carbon Materials by the Yolk-Shell
Design. J. Am. Chem. Soc..

[19] Du X., Guan G., Li X., Jagadale A. D., Ma X., Wang Z., Hao X., Abudula A. (2016). A novel electroactive λ-MnO_2_/PPy/PSS
core–shell nanorod coated electrode for selective recovery
of lithium ions at low concentration. J. Mater.
Chem. A.

[20] Sun C., Zhao B., Mao J., Dai K. H., Wang Z. y., Tang L. B., Chen H. z., Zhang X. H., Zheng J. C. (2023). Enhanced
Cycling Stability of 4.6 V LiCoO_2_ Cathodes by Inhibiting
Catalytic Activity of its Interface Via MXene Modification. Adv. Funct. Mater..

[21] Yuan Y., Han L., Wang N. (2022). Selective
recovery of palladium from nuclear waste
by covalent organic framework. Matter.

[22] Ban J., Xu H., Cao G., Fan Y., Pang W. K., Shao G., Hu J. (2023). Synergistic
Effects of Phase Transition and Electron-Spin Regulation
on the Electrocatalysis Performance of Ternary Nitride. Adv. Funct. Mater..

[23] Feng L., Wang H., Liu T., Feng T., Cao M., Zhang J., Liu T., Guo Z., Galiotis C., Yuan Y., Wang N. (2023). Ultrasensitive and
highly selective detection of strontium ions. Nat. Sustainability.

[24] Yang S., Wang Y., Pan H., He P., Zhou H. (2024). Lithium extraction
from low-quality brines. Nature.

