# Supplementary material for: Activating Lithium Titanate for High-Performance and Stable Electrochemical Direct Lithium Extraction
Source: Environ Sci Technol. 2026 Jan 16;60(4):3681–92. doi: 10.1021/acs.est.5c14846 (PMC12874507; doi:10.1021/acs.est.5c14846)
Supplement: Supplementary file 1 [file es5c14846_si_001.pdf]

# Supplementary Information

# Activating lithium titanate for high-performance and stable electrochemical direct lithium extraction

Bing Zhao,[a,b#] Longqian Xu,[b#] Yingjun Qiao,[a] Zhiqiang Qian,[a] Wenfei Wei,[a] Xudong Zhang,[b] ,Zhong Liu,[a*] and Shihong Lin[b,c*]

[a] Key Laboratory of Green and High-end Utilization of Salt Lake Resources, Qinghai Institute of Salt Lakes, Qinghai Province Key Laboratory of Resources and Chemistry of Salt Lakes, Chinese Academy of Sciences, Xining, Qinghai 810008, China

[b] Department of Civil and Environmental Engineering, Vanderbilt University, Nashville, Tennessee 37235-1831, United States

[c] Department of Chemical and Biomolecular Engineering, Vanderbilt University, Nashville, Tennessee 37235-1604, United States

*Corresponding author

#These authors contribute equally.

(*Email： liuzhong@isl.ac.cn and shihong.lin@vanderbilt.edu)

**The PDF file includes:**

Supporting Methods

Supporting Figures 1-11

Supporting Tables 1-8

Supporting References

23

# Table of Contents

1. Calculation of separation factors ....................................................4

2. Supplemental Figures....................................................5

3. Supplemental Tables....................................................12

References ....................................................17

## Text S1. Calculation of separation factors

The separation factor ($\alpha_{Li/M}$) quantitatively describes the preferential uptake of $Li^+$ over a competing cation $M^+$ (where $M^+$ = $Na^+$, $K^+$, $Mg^{2+}$, or $Ca^{2+}$) during the electrosorption process. It is calculated based on the change in solution composition, comparing the ionic ratios in the feed brine and the receiving solution (eluate) collected after the desorption step.

The separation factor is defined as the ratio of the $Li^+$-to-$M^+$ molar concentration ratio in the receiving solution to that in the feed brine:

$$\alpha_{\text{Li/M}} = \frac{(C_{\text{Li}}/C_{\text{M}})_{\text{receiving}}}{(C_{\text{Li}}/C_{\text{M}})_{\text{feed}}}$$

where$C_{\text{Li}}$and$C_{\text{M}}$are the molar concentrations of lithium and the competing ion, respectively.

1.1 Step-by-Step Calculation Example (for $\alpha_{Li/Na}$):

Measure Concentrations: The concentrations of $Li^+$ and $Na^+$ are measured by ICP-OES in both the feed brine and the receiving solution.

Feed brine: $C_{\text{Li, feed}} = 6.0$ mM, $C_{\text{Na, feed}} = 1200$ mM

Receiving solution: $C_{\text{Li, receiving}} = 48.0$ mM, $C_{\text{Na, receiving}} = 137.10$ mM

Calculate Molar Ratios:

In feed brine:$(C_{\text{Li}}/C_{\text{Na}})_{\text{feed}} = (6.0/1200) = 0.005$

In receiving solution:$(C_{\text{Li}}/C_{\text{Na}})_{\text{receiving}} = (48.0/137.1) \approx 0.35$

Compute Separation Factor:

$$\alpha_{\text{Li/Na}} = \frac{0.350}{0.005} = 70.0$$

An $\alpha_{Li/Na}$ value of 70.0 indicates that the $Li^+/Na^+$ ratio in the receiving solution is 70 times higher than that in the feed brine, demonstrating high selectivity for $Li^+$ over $Na^+$.

## 2. Supplemental Figures

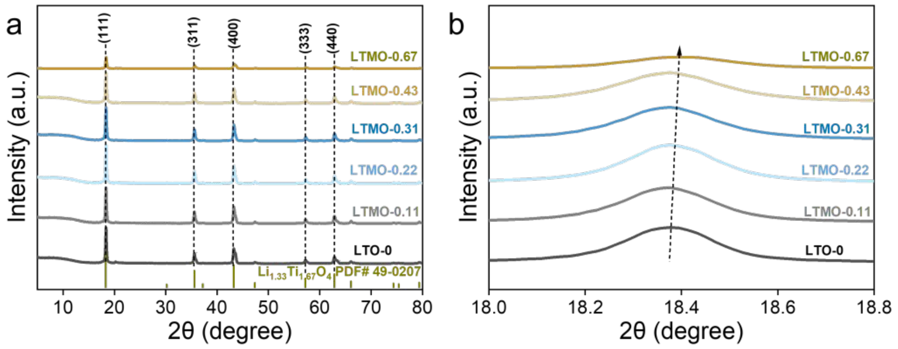


**Figure S1. Structural evolution of LTMO-R samples with increasing Mn substitution.** (a) X-ray diffraction (XRD) patterns of $Li_{1.33}Ti_xMn_yO_4$ (LTMO-R) samples with increasing Mn content, corresponding to R = 0 ($Ti_{1.67}$), 0.11, 0.22, 0.31, 0.43, and 0.67. (b) Enlarged view of the (111) diffraction peak highlighting a gradual rightward shift upon Mn substitution, indicating lattice contraction due to smaller Mn ionic radius. These results confirm successful incorporation of Mn into the spinel lattice.

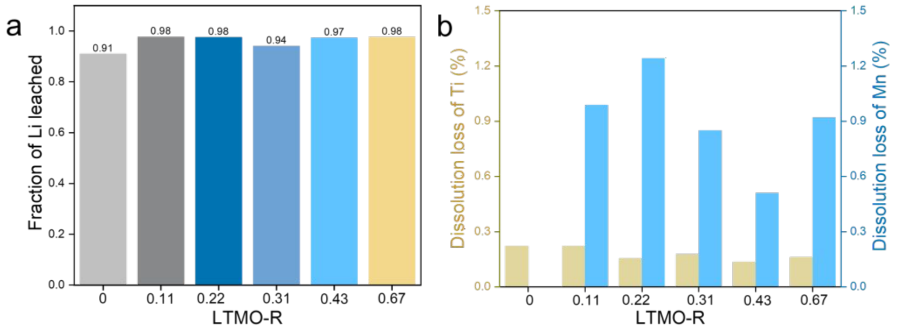


**Figure S2. Delithiation and framework stability were evaluated via acid leaching under mild conditions (0.1 mol/L HCl, solid–liquid ratio = 1 g/L, 24 h)**. (a) Percentage of Li leached from the crystals; (b) Leaching of Ti and Mn from LTMO-R particles with increasing Mn substitution. The tested samples included $Li_{1.33}Ti_{1.67}O_4$ (R = 0), $Li_{1.33}Ti_{1.5}Mn_{0.17}O_4$ (R = 0.11), $Li_{1.33}Ti_{1.37}Mn_{0.3}O_4$ (R = 0.22), $Li_{1.33}Ti_{1.26}Mn_{0.41}O_4$ (R = 0.31), $Li_{1.33}Ti_{1.17}Mn_{0.5}O_4$ (R = 0.43), and $Li_{1.33}Ti_{1.00}Mn_{0.67}O_4$ (R = 0.67), respectively. These results suggest that Li can be removed from LTMO without considerable impact on the crystal stability.

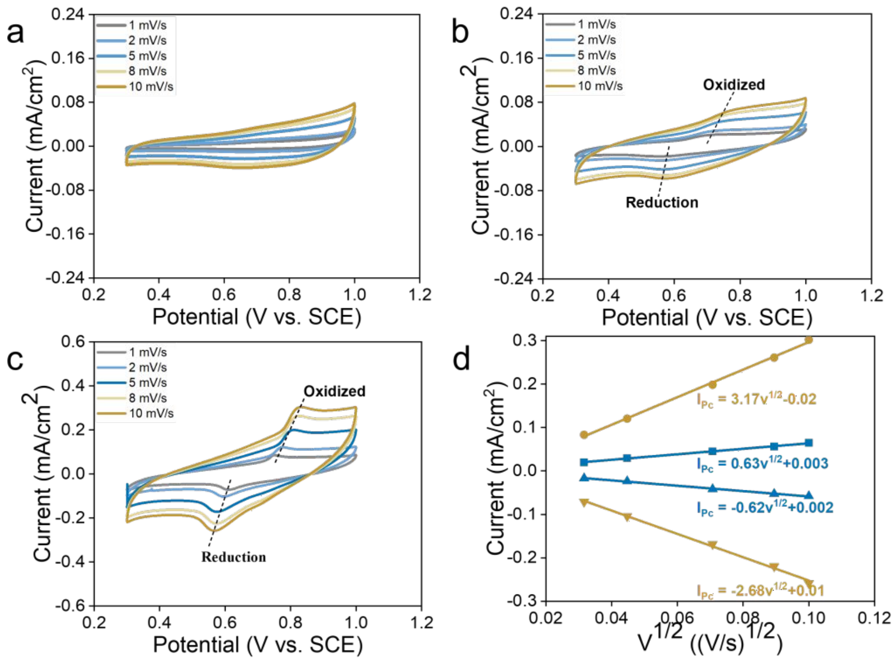


**Figure S3. Cyclic voltammetry (CV) curves** of (a) $H_{1.33}Ti_{1.67}O_4$, (b) $H_{1.33}Ti_{1.37}Mn_{0.3}O_4$, and (c) $H_{1.33}Ti_{1.17}Mn_{0.5}O_4$ recorded in 1.0 mol $L^{-1}$ LiCl at room temperature using a three-electrode setup (working electrode: HTO/HTMO@CNT@PVDF film, counter electrode: carbon, reference electrode: SCE). CVs were collected at scan rates ranging from 1 to 10 mV/s within the potential window of 0.3–1.1 V vs. AgCl. (d) Linear relationships between anodic ($I_{pa}$) and cathodic ($I_{pc}$) peak currents and the square root of scan rate ($v^{1/2}$) for Mn-substituted electrodes ($H_{1.33}Ti_{1.37}Mn_{0.3}O_4$ and $H_{1.33}Ti_{1.17}Mn_{0.5}O_4$). Only redox-active materials are shown, as the undoped $H_{1.33}Ti_{1.67}O_4$ lacks observable redox peaks. The linear dependence suggests a diffusion-controlled redox mechanism associated with $Mn^{3+}/Mn^{4+}$ couples.

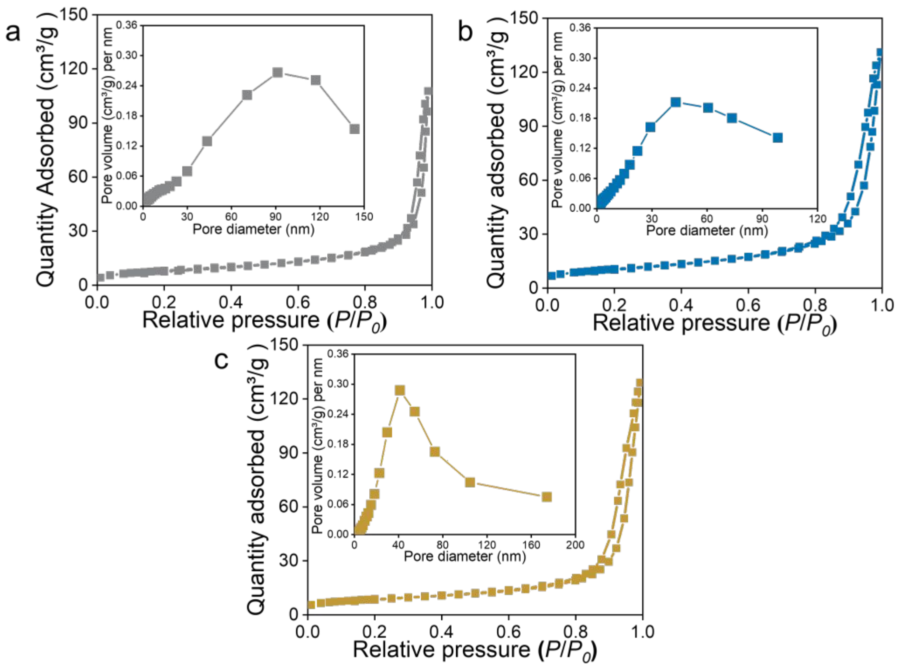


**Figure S4. BET Surface area analysis of HTO and HTMO particles.** $N_2$ adsorption–desorption isotherms and corresponding BJH pore size distributions (insets) for (a) $H_{1.33}Ti_{1.67}O_4$, (b) $H_{1.33}Ti_{1.37}Mn_{0.3}O_4$, and (c) $H_{1.33}Ti_{1.17}Mn_{0.5}O_4$, respectively. The adsorption volume (cm³ /g, STP) increases with Mn content, indicating an enlarged accessible surface area. Pore size distributions show a shift toward smaller diameters and increased pore volume, suggesting enhanced microporosity. These structural features contribute to improved electrolyte accessibility and $Li^+$ transport kinetics. BET surface areas were calculated as 40.08, 43.56, and 52.64 $m^2/g$ for the three samples, respectively.

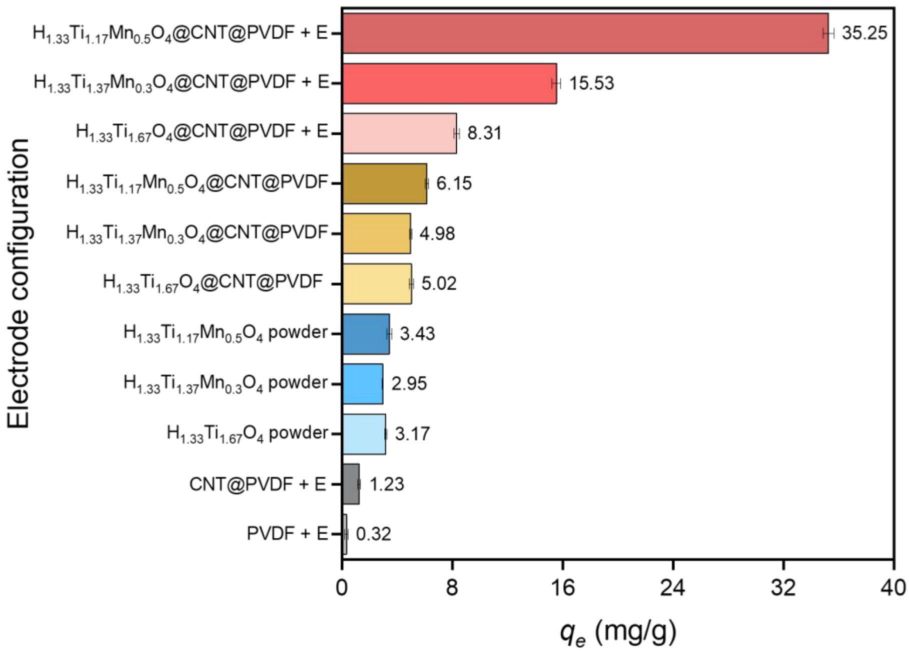


**Figure S5. Equilibrium adsorption capacity for different adsorbents with and without applied voltage**. Equilibrium $Li^+$ adsorption capacity of $H_{1.33}Ti_{1.67}O_4$, $H_{1.33}Ti_{1.37}Mn_{0.3}O_4$, and $H_{1.33}Ti_{1.17}Mn_{0.5}O_4$ in 166.67 mg/L LiCl solutions were compared under different configurations: powder form (no voltage), CNT@PVDF composite film without applied voltage, and adsorbent particle-loaded CNT@PVDF electrodes with an applied voltage of 1.2 V (+E). Also evaluated were the capacity of the additives (PVDF/CNT) in making the electrodes with and without applied voltage.

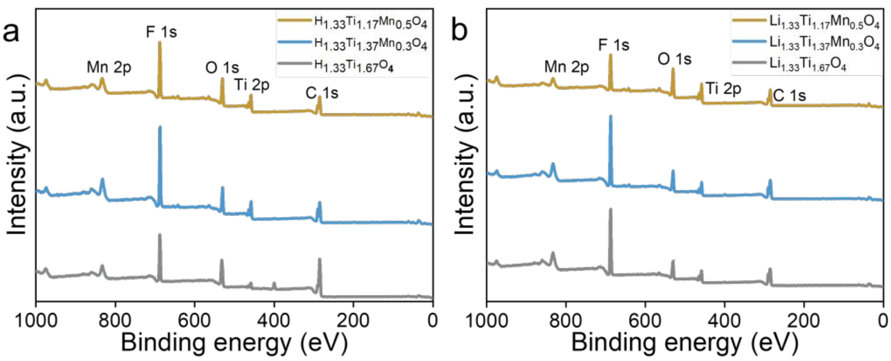


**Figure S6. Surface chemical composition of HTMO after electrochemical cycling.** Survey X-ray photoelectron spectroscopy (XPS) spectra of electrodes (a) before and (b) after electrosorption, revealing elemental states and surface composition changes associated with $Li^+$ uptake. The absence of significant peak shifts or intensity loss suggests that no substantial elemental dissolution or contamination occurred during the process.

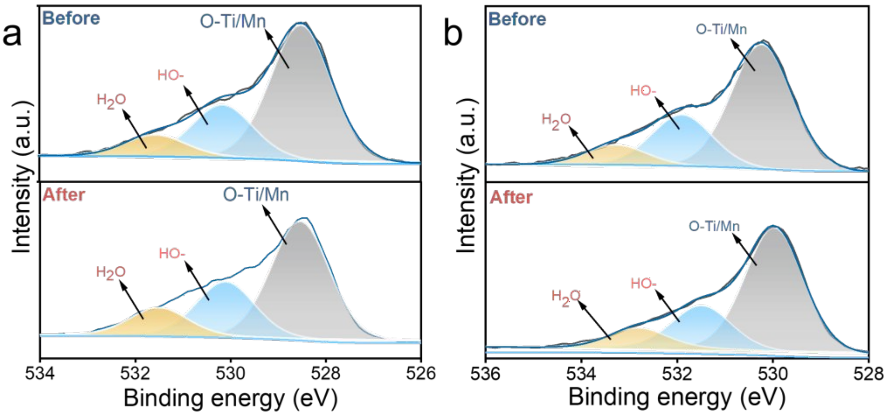


**Figure S7. High-resolution XPS analysis of oxygen coordination environments.** O 1s spectra of (a) $H_{1.33}Ti_{1.37}Mn_{0.3}O_4$ and (b) $H_{1.33}Ti_{1.17}Mn_{0.5}O_4$ before and after $Li^+$ adsorption. Deconvoluted peaks reveal changes in lattice oxygen (O–Ti/Mn), surface hydroxyl groups (–OH), and adsorbed water ($H_2O$), reflecting local coordination rearrangements associated with redox-coupled $Li^+$ intercalation.

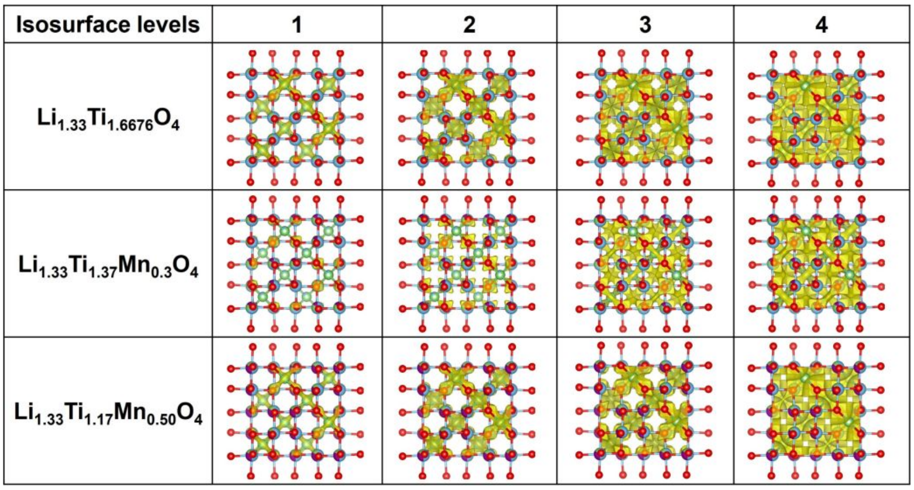


**Figure S8. $Li^+$ Bond-valence-based $Li^+$ migration pathways in HTMO structures.** BV isosurfaces of $Li_{1.33}Ti_{1.67}O_4$, $Li_{1.33}Ti_{1.37}Mn_{0.3}O_4$, and $Li_{1.33}Ti_{1.17}Mn_{0.5}O_4$ across four energy levels reveal the evolution of percolation networks and $Li^+$ transport accessibility as a function of Mn substitution. $Li_{1.33}Ti_{1.37}Mn_{0.3}O_4$ shows limited connectivity at low iso-surface levels, continuous migration networks only emerge at higher thresholds, suggesting that strong driving forces (e.g., high voltage) are needed to activate diffusion. In contrast, $Li_{1.33}Ti_{1.17}Mn_{0.5}O_4$ exhibits well-connected pathways even at low thresholds, indicating lower activation barriers for $Li^+$ transport and better electrochemical accessibility under mild conditions.

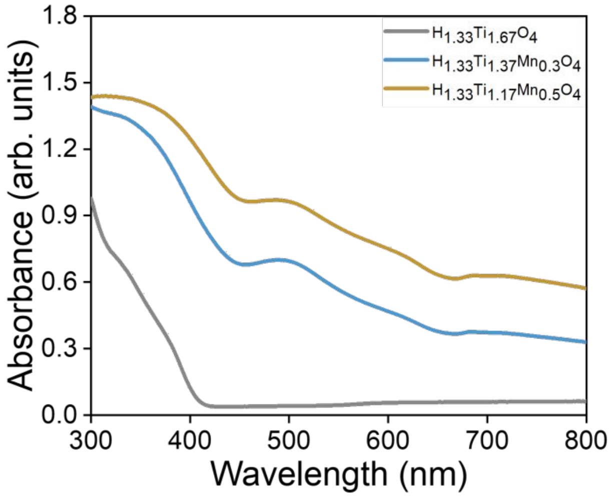


**Figure S9. Optical band gap modulation via Mn substitution**. UV–Vis diffuse reflectance spectra of $H_{1.33}Ti_{1.67}O_4$, $H_{1.33}Ti_{1.37}Mn_{0.3}O_4$, and $H_{1.33}Ti_{1.17}Mn_{0.5}O_4$, showing progressive redshift in absorption edge with increasing Mn content. Mn content induces a redshift in the absorption edge, indicating progressive band gap narrowing. This spectral shift suggests enhanced electronic delocalization within the lattice, which may facilitate charge transport and support the improved electrochemical performance observed in Mn-rich compositions.

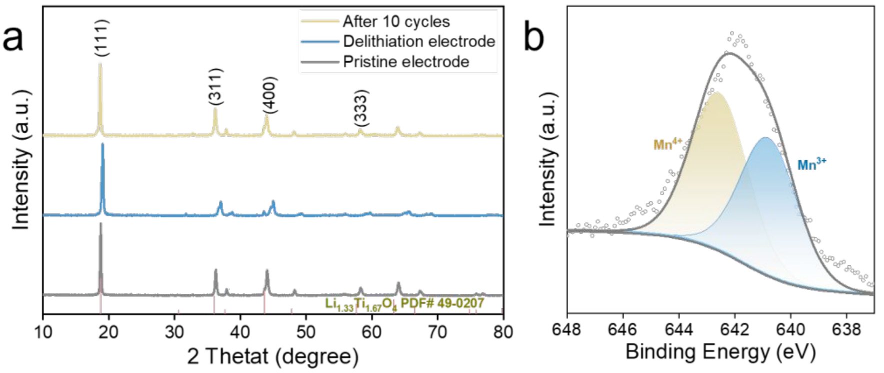


**Figure S10. Structural and chemical stability of Ti-stabilized HTMO electrodes after cycling.** (a) Ex-situ XRD patterns of pristine, delithiated, and cycled (10 cycles) $H_{1.33}Ti_{1.17}Mn_{0.5}O_4$ electrodes, confirming full retention of the spinel structure with no formation of secondary phases. (b) High-resolution Mn 2p XPS spectra of cycled electrodes, showing that the $Mn^{3+}/Mn^{4+}$ ratio remains nearly unchanged after 10 charge/discharge cycles. This result verifies the reversible operation of the Mn redox couple and the absence of irreversible oxidation or reduction during cycling.

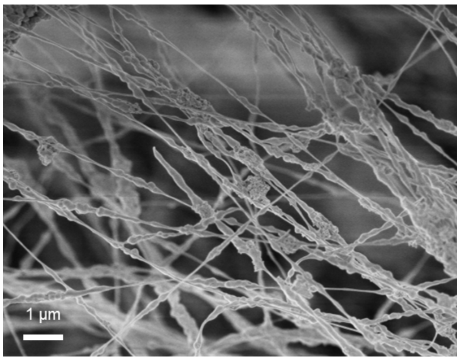


**Figure S11. Morphological stability of HTMO electrodes after cycling.** SEM image of the electrospun $H_{1.33}Ti_{1.17}Mn_{0.5}O_4$ electrode after 10 charging–discharging cycles with pretreated (0.22 μm microfiltration) real Lagoco brine, showing complete retention of the interconnected fibrous network without surface fouling or pore blockage. The preserved morphology indicates minimal structural perturbation during operation and supports the short-term cycling stability demonstrated in Mn-substituted HTMO.

## 3. Supplemental Tables

**Table S1.** Performance comparison of manganese oxides electrodes and this work.

| Doping element | Electrodes | Doping amuont | Volatege/ Current (V/mA) | $C_0$ | $q_e$ (mg/g) | Ref |
|---|---|---|---|---|---|---|
| Sr | $LiMn_{1.75}Sr_{0.25}O_4$ | Sr/Mn=0.14 | 1 | 138.92 | 30.12 | [1] |
| Ni | LNMO-MS(0.05) | Ni/Mn=0.10 | 1 | 380 | 28.52 | [2] |
| La | LLMO-2 | La/Mn=0.04 | 1 | 350 | 23.5 | [3] |
| Bi | LMBO-2 | Bi/Mn=0.02 | 50 | 417 | 30 | [4] |
| Cr | $LiMn_{1.8}Cr_{0.2}O_4$ | Cr/Mn=0.2 | 1.1 | 320 | 21.85 | [5] |
| N | C-N@LMO | N/Mn=0.2 | 1 | 200 | 22.06 | [6] |
| Al | $LiAl_{0.05}Mn_{1.95}O_4$ | N/Mn=0.03 | 1 | 400 | 29 | [7] |
| Ti | $H_4Mn_{3.5}Ti_{1.5}O_{12}$ | Ti/Mn=0.4 | --- | 134.7 | 18 | [8] |
| Mn | LTO | Ti = 100%<br>Mn:Ti= 0.21<br>Mn:Ti = 0.42 | 1.2 | 166.17 | 6.56<br>16.56<br>35.56 | This work |

**Table S2.** EDS element table of materials after introduction of electrically active Mn with different contents.

| Materials | O ( wt% ) | Ti ( wt% ) | Mn ( wt% ) | $n_{Mn}:n_{total}$ | Molecular formula |
|---|---|---|---|---|---|
| 0 | 66.3 | 33.7 | 0 | 0 | $Li_{1.33}Ti_{1.67}O_4$ |
| 1 | 66.6 | 39.8 | 3.7 | 0.08 | $Li_{1.33}Ti_{1.54}Mn_{0.13}O_4$ |
| 2 | 67.2 | 27.2 | 5.6 | 0.15 | $Li_{1.33}Ti_{1.37}Mn_{0.3}O_4$ |
| 3 | 65.5 | 25.2 | 9.3 | 0.25 | $Li_{1.33}Ti_{1.26}Mn_{0.41}O_4$ |
| 4 | 64.4 | 25.6 | 10 | 0.30 | $Li_{1.33}Ti_{1.17}Mn_{0.5}O_4$ |
| 5 | 63.5 | 21.5 | 15 | 0.38 | $Li_{1.33}Ti_{1.03}Mn_{0.64}O_4$ |

**Table S3.** Evolution of local bond lengths with increasing Mn content in $Li_{1.33}Ti_yMn_xO_4$.

| | Li-O (Å) | Ti-O (Å) | Mn-O (Å) | a = b = c (Å) |
|---|---|---|---|---|
| LTO | 1.989 | 1.992 | - | 8.357 |
| $Li_{1.33}Ti_{1.37}Mn_{0.3}O_4$ | 1.986 | 1.991 | 1.989 | 8.352 |
| $Li_{1.33}Ti_{1.17}Mn_{0.5}O_4$ | 1.983 | 1.996 | 1.992 | 8.363 |
| LMO | 1.969 | - | 1.916 | 8.122 |

*The pristine LTO (x=0) serves as the baseline. A systematic decrease in Li-O bond length and an increase in Ti-O bond length at higher doping (x=0.5) are observed, indicating local lattice distortion.

**Table. S4.** Electrochemical impedance parameters demonstrating the enhanced kinetics of Mn-doped HTMO electrodes compared to the undoped baseline.

| Materials | $R_s$ (Ω) | $R_{ct}$ (Ω) | W (Ω) | $D_{Li^+}$ ($cm^2/s$) |
|---|---|---|---|---|
| $H_{1.33}Ti_{1.67}O_4$ | 3.72 | 5.26 | 11.60 | $1.54*10^{-13}$ |
| $H_{1.33}Ti_{1.37}Mn_{0.3}O_4$ | 4.73 | 0.96 | 27.11 | $5.25*10^{-13}$ |
| $H_{1.33}Ti_{1.17}Mn_{0.5}O_4$ | 4.09 | 2.96 | 2.90 | $2.15*10^{-11}$ |
| LMO | 4.14 | 1.62 | 24.1 | $3.97*10^{-11}$ |

**Table. S5.** BET surface area and pore structural parameters of the Ti–Mn spinel series.

| Materials | Surface Area ($m^2/g$) | Pore diameter | |
|---|---|---|---|
| | | Adsorption (nm) | Desorption (nm) |
| $H_{1.33}Ti_{1.67}O_4$ | 40.08 | 22.70 | 26.18 |
| $H_{1.33}Ti_{1.37}Mn_{0.3}O_4$ | 45.93 | 32.07 | 29.31 |
| $H_{1.33}Ti_{1.17}Mn_{0.5}O_4$ | 52.64 | 20.77 | 21.12 |

**Table S6.** Comparison of separation performance with traditional adsorbents.

| Separation materials | pH | $C_0$ (mg/L) | $q_t$@1h (mg/g) | $q_e$ (mg/g) | $t$ (min) | $R_{60}$ min (mg/g/min) | Ref |
|---|---|---|---|---|---|---|---|
| LiAl-LDH | 7 | 750 | 5.83 | 6.02 | 180 | 0.10 | [9] |
| LiAl-LDH | 8 | 1000 | 9 | 9.12 | 90 | 0.15 | [10] |
| LiAl-LDH | 6 | 500 | 7 | 9.66 | 400 | 0.12 | [11] |
| LiAl-LDH-$SO_4$ | 8 | 138.82 | 3.89 | 4.16 | 120 | 0.065 | [12] |
| $HMn_2O_4$ | --- | 73 | 7.41 | 15.66 | 36000 | 0.10 | [13] |
| $HMn_2O_4$ | --- | 73 | 7.22 | 10.82 | 28800 | 0.12 | |
| $H_4Mn_5O_{12}$ | --- | | 4.25 | 16.50 | 10800 | 0.071 | [14] |
| $H_4Mn_5O_{12}$ | 12 | 60 | 4.12 | 12.31 | 72000 | 0.07 | [15] |
| $H_4Mn_5O_{12}$ | 12 | 185.8 | 17.21 | 22.52 | 500 | 0.28 | [16] |
| $H_{1.6}Mn_{1.6}O_4$ | 12 | | 8.25 | 32.3 | 2000 | 0.12 | [17] |
| $H_{1.6}Mn_{1.6}O_4$-Na | 12 | 249.88 | 9.25 | 33.35 | 1500 | 0.15 | [18] |
| $H_{1.6}Mn_{1.6}O_4$-Al | 12 | 249.88 | 10.21 | 29.40 | 2000 | 0.17 | [19] |
| $H_{1.6}Mn_{1.6}O_4$-F | 12 | 249.88 | 12.56 | 33.33 | 2400 | 0.21 | [20] |
| $H_{1.6}Mn_{1.6}O_4$ | 9.5 | | 15.23 | 26.21 | 14400 | 0.25 | [21] |
| $H_2TiO_3$ | 12.3 | | 25.61 | 31.88 | 360 | 0.43 | [22] |
| $H_2TiO_3$ | 12 | | 25.52 | 28.89 | 240 | 0.43 | [23] |
| $H_2TiO_3$ | 12 | | 24.21 | 29.11 | 300 | 0.40 | [24] |
| $H_4Ti_5O_{12}$ | 12.75 | 249.88 | 21.20 | 22.95 | 180 | 0.35 | [25] |
| $H_4Ti_5O_{12}$ | 12.75 | 249.88 | 18.21 | 25.85 | 180 | 0.30 | [26] |
| $H_4Ti_5O_{12}$ | 12. | 200 | 20 | 28.12 | 240 | 0.33 | [27] |

| | | | | | | | |
|---|---|---|---|---|---|---|---|
| | 3 | | | | | | |
| $HMn_2O_4$+E | --- | | 13.10 | 18.25 | 300 | 0.22 | [28] |
| $HMn_2O_4$+E | --- | | 24.26 | 32.22 | 180 | 0.40 | [29] |
| $HMn_2O_4$+E | --- | 100 | 20.85 | 22.56 | 180 | 0.34 | [30] |
| LFPO (100) | 7 | 50 | 3.02 | 5.66 | 180 | 0.050 | [31] |
| LFPO (010) | 7 | 50 | 2.21 | 11.12 | 360 | 0.037 | |
| LFPO | 8 | 100 | 2.86 | 2.92 | 60 | 0.048 | [32] |
| $H_{1.33}Ti_{1.17}Mn_{0.5}O_4$ | 9 | | 25.56 | 43.58 | 180 | 0.43 | This work |

**Table S7.** Comparison of the performance of electrochemical lithium extraction.

| Electrode materials | Adsorption Capacity (mg/g) | Li/Na | Li/Mg | Capacity retention rate/n (cycles)* | Dissolution of Mn/Fe | Ref |
|---|---|---|---|---|---|---|
| LNMO/AC | 1.80 | 11 | 167 | --- | --- | 33 |
| LMO-Al | 21.70 | 80 | 100 | --- | 1.85% | 34 |
| $LiMn_2O_4/AlF_3$ | 31.50 | 2.6 | 1.8 | --- | 1.98% | 35 |
| LFPO (100) | 11.13 | 20 | 18 | 96.50%/5 | --- | 31 |
| LFPO (010) | 19.98 | 14 | 13.22 | 80.11%/5 | --- | |
| LFPO | 10.10 | --- | --- | 42.86%/5 | --- | 32 |
| $HMn_2O_4$/AC | 24.56 | 106.85 | --- | 80.10%/5 | 3.53% | 36 |
| $H_{1.6}Mn_{1.6}O_4$/AC | 34.40 | 117.92 | --- | 86.21%/5 | 2.21 | |
| λ-$MnO_2$/PPy/PSS | 35.2 | 46 | --- | 95.55% | --- | 37 |
| HMO | 31.1 | 29.44 | 35.60 | 81.63%/5 | 0.52% | 38 |
| HMO/rGO-s | 35.9 | 37.83 | 49.22 | 91.27%/5 | 0.35% | |
| $LiMn_2O_4$ | 22.7 | 88 | --- | --- | --- | 39 |
| λ-$MnO_2$ | 14 | 38.78 | 29.04 | 82.8%/50 | --- | 40 |
| $FePO_4$ | 38.93 | --- | 7.26 | 87%/31 | | 41 |
| $H_{1.6}Mn_{1.6}O_4$ | 38.78 | 10.39 | 10.23 | --- | --- | 42 |
| $Li_{1-x}Mn_2O_4$ | 34.1 | 300 | 70 | --- | --- | 43 |
| $H_{1.33}Ti_{1.17}Mn_{0.5}O_4$ | 43.58 | 420.56 | 56.19 | 95.63%/5 | 0.43% | This work |

**Table S8.** The main composition of ions in Lagoco Salt Lake.

| Composition | $Li^+$ (mg/L) | $Na^+$ (mg/L) | $K^+$ (mg/L) | $Mg^{2+}$ (mg/L) | $Ca^{2+}$ (mg/L) |
|---|---|---|---|---|---|
| Lagoco | 255.01 | 16719.56 | 2445.21 | 787.482 | 38.47 |

## References


(1) Fang, D.; Zhang, X.; Liu, F.; Hu, S.; Zhang, P.; Li, K. Structural stabilization via strontium doping in $LiMn_2O_4$ enables selective lithium capture from high-mg brines by rocking-chair capacitive deionization. *Chem. Eng. J.* **2025**, *521*, 166161-166169. DOI: 10.1016/j.cej.2025.166161.

(2) Wei, Z.; Hu, B.; Yao, C.; Yang, J.; Zhang, B.; Wang, Y.; Li, X.; Guo, J.; Liu, J. Dual-engineered Ni-$LiMn_2O_4$ microsheets for sustainable lithium mining: Accelerated ion transport and robust electrochemical extraction in brine. *J. Colloid Interface Sci.* **2025**, *693*, 137655-137667. DOI: 10.1016/j.jcis.2025.137655.

(3) Ban, J.; Xu, H.; Cao, G.; Fan, Y.; Pang, W. K.; Shao, G.; Hu, J. Synergistic Effects of Phase Transition and Electron-Spin Regulation on the Electrocatalysis Performance of Ternary Nitride. *Adv. Funct. Mater.* **2023**, *33* (25), 2300623-2300634. DOI: 10.1002/adfm.202300623.

(4) Gou, L.; Zhang, Y.-F.; Wang, W.; Ying, J.-Y.; Fan, X.-Y.; Zhang, Z.-Z. Triple synergistic effects of bismuth doping in spinel-$LiMn_2O_4$: A path to high-performance electrochemical lithium extraction from salt-lake brine. *Chem. Eng. J.* **2024**, *498*, 155755-155765. DOI: 10.1016/j.cej.2024.155755.

(5) Tian, G.; Gao, J.; Wang, M.; Wen, X.; Liu, Y.; Xiang, J.; Zhang, L.; Cheng, P.; Zhang, J.; Tang, N. Structural stabilization of Cr-doped spinel $LiMn_2O_4$ for long-term cyclability towards electrochemical lithium recovery in original brine. *Electrochim. Acta* **2024**, *475*, 143361-143371. DOI: 10.1016/j.electacta.2023.143361.

(6) DuChanois, R. M.; Cooper, N. J.; Lee, B.; Patel, S. K.; Mazurowski, L.; Graedel, T. E.; Elimelech, M. Prospects of metal recovery from wastewater and brine. *Nature Water* **2023**, *1* (1), 37-46. DOI: 10.1038/s44221-022-00006-z.

(7) Tang, J.; Luo, Q.; Wu, Z.; Shi, K. Single-crystalline Al-doped $LiMn_2O_4$ nanotubes for electrochemical lithium extraction from brines. *Chem. Eng. J.* **2025**, *505*, 159256-159266. DOI: 10.1016/j.cej.2025.159256.

(8) Cui, J.; Xu, H.; Ding, Y.; Tian, J.; Zhang, X.; Jin, G. Recovery of lithium using $H_4Mn_{3.5}Ti_{1.5}O_{12}$/reduced graphene oxide/polyacrylamide composite hydrogel from brine by Ads-ESIX process. *Chinese Journal of Chemical Engineering* **2022**, *44*, 20-28. DOI: 10.1016/j.cjche.2021.05.009.

(9) Zhang, L.; Zhang, T.; Zhao, Y.; Dong, G.; Lv, S.; Ma, S.; Song, S.; Quintana, M. Doping engineering of lithium-aluminum layered double hydroxides for high-efficiency lithium extraction from salt lake brines. *Nano Research* **2023**, *17* (3), 1646-1654. DOI: 10.1007/s12274-023-5950-1.

(10) Lv, S.; Zhao, Y.; Zhang, L.; Zhang, T.; Dong, G.; Li, D.; Cheng, S.; Ma, S.; Song, S.; Quintana, M. Anion regulation strategy of lithium-aluminum layered double hydroxides for strengthening resistance to deactivation in lithium recovery from brines. *Chemical Engineering Journal* **2023**, *472*. DOI: 10.1016/j.cej.2023.145026.

(11) Qinglong Luo; Mingzhe Dong; Guoliang Nie; Zhong Liu; Zhijian Wu; Li, J. Extraction of lithium from salt lake brines by granulated adsorbents. *Colloids and Surfaces A: Physicochemical and Engineering Aspects* **2021,**, *628*, 127256-127265.

(12) Chen, J.; Yuan, H.; Yu, J.; Yan, M.; Yang, Y.; Lin, S. Regulating lithium extraction based on intercalated $SO_4^{2-}$ in Li/Al-LDHs. *Journal of Colloid And Interface Science* **2023**, *649*, 694-702. DOI: 10.1016/j.jcis.2023.06.165.

(13) Wajima, T.; Munakata, K.; Uda, T. Adsorption Behavior of Lithium from Seawater using Manganese Oxide Adsorbent. *Plasma and Fusion Research* **2012**, *7* (0), 2405021-2405021. DOI: 10.1585/pfr.7.2405021.

(14) Tian, L.; Liu, Y.; Tang, P.; Yang, Y.; Wang, X.; Chen, T.; Bai, Y.; Tiraferri, A.; Liu, B. Lithium extraction from shale gas flowback and produced water using $H_{1.33}Mn_{1.67}O_4$ adsorbent. *Resources, Conservation and Recycling* **2022**, *185*. DOI: 10.1016/j.resconrec.2022.106476.

(15) Ding, W.; Zhang, J.; Liu, Y.; Guo, Y.; Deng, T.; Yu, X. Synthesis of granulated $H_4Mn_5O_{12}$/chitosan with improved stability by a novel cross-linking strategy for lithium adsorption from aqueous solutions. *Chemical Engineering Journal* **2021**, *426*, 131689-131697. DOI: 10.1016/j.cej.2021.131689.

(16) Xu, N.; Li, S.; Guo, M.; Qian, Z.; Li, W.; Liu, Z. Synthesis of $H_4Mn_5O_{12}$ Nanotubes Lithium Ion Sieve and Its Adsorption Properties for $Li^+$ from Aqueous Solution. *ChemistrySelect* **2019**, *4* (33), 9562-9569. DOI: 10.1002/slct.201901764.

(17) Qian, F.; Zhao, B.; Guo, M.; Qian, Z.; Xu, N.; Wu, Z.; Liu, Z. Enhancing the Li+ adsorption and anti-dissolution properties of Li1.6Mn1.6O4 with Fe, Co doped. *Hydrometallurgy* **2020**, *193*. DOI: 10.1016/j.hydromet.2020.105291.

(18) Qian, F.; Zhao, B.; Guo, M.; Wu, Z.; Zhou, W.; Liu, Z. Surface trace doping of Na enhancing structure stability and adsorption properties of $Li_{1.6}Mn_{1.6}O_4$ for $Li^+$ recovery. *Separation and Purification Technology* **2021**, *256*, 117583-117592. DOI: 10.1016/j.seppur.2020.117583.

(19) Qian, F.; Guo, M.; Qian, Z.; Zhao, B.; Li, J.; Wu, Z.; Liu, Z. Enabling highly structure stability and adsorption performances of $Li_{1.6}Mn_{1.6}O_4$ by Al-gradient surface doping. *Separation and Purification Technology* **2021**, *264*, 118433-118442. DOI: 10.1016/j.seppur.2021.118433.

(20) Qian, F.; Zhao, B.; Guo, M.; Qian, Z.; Wu, Z.; Liu, Z. Trace doping by fluoride and sulfur to enhance adsorption capacity of manganese oxides for lithium recovery. *Materials & Design* **2020**, *194*. DOI: 10.1016/j.matdes.2020.108867.

(21) Qi, S.; Xu, L.; Shao, H.; Huang, C.; Ma, L.; Han, J.; Xue, X.; Zhang, M.; Ma, H. Cross-linked HMO/PVA nanofiber mats for efficient lithium extraction from Salt-lake. *Separation and Purification Technology* **2023**, *323*. DOI: 10.1016/j.seppur.2023.124382.

(22) Sun, J.; Li, X.; Huang, Y.; Luo, G.; Tao, D.; Yu, J.; Chen, L.; Chao, Y.; Zhu, W. Preparation of high hydrophilic $H_2TiO_3$ ion sieve for lithium recovery from liquid lithium resources. *Chemical Engineering Journal* **2023**, *453*,

139485-139495. DOI: 10.1016/j.cej.2022.139485.

(23) Dai, X.; Zhan, H.; Qian, Z.; Li, J.; Liu, Z.; Wu, Z. Al-doped H(2)TiO(3) ion sieve with enhanced Li(+) adsorption performance. *RSC Adv* **2021**, *11* (55), 34988-34995. DOI: 10.1039/d1ra06535a.

(24) Qiao, Y.; Dai, X.; Zhao, B.; Qian, Z.; Wu, Z.; Liu, Z. Enhanced lithium extraction from aqueous solutions using multi-scale modified titanium adsorbent. *Separation and Purification Technology* **2024**, *351*. DOI: 10.1016/j.seppur.2024.128060.

(25) Zhao, B.; Qian, Z.; Qiao, Y.; Li, J.; Wu, Z.; Liu, Z. The $Li(H_2O)_n$ dehydration behavior influences the $Li^+$ ion adsorption on $H_4Ti_5O_{12}$ with different facets exposed. *Chemical Engineering Journal* **2023**, *451*, 138870. DOI: 10.1016/j.cej.2022.138870.

(26) Zhao, B.; Qiao, Y.; Qian, Z.; Wei, W.; Li, J.; Wu, Z.; Liu, Z. Unraveling the $Li^+$ desorption behavior and mechanism of $Li_4Ti_5O_{12}$ with different facets to enhance lithium extraction. *Journal of Materials Chemistry A* **2023**, *11* (13), 7094-7104. DOI: 10.1039/d2ta10016a.

(27) Xiao, X.; Li, J.; Qiu, K.; Chen, M.; Xie, H. Synergistic effect of Fe/Zr dual doping endows hydrophilic spinel-structured $H_4Ti_5O_{12}$ ion sieves for efficient lithium extraction from liquid resources. *Separation and Purification Technology* **2025**, *358*, 130358-130370. DOI: 10.1016/j.seppur.2024.130358.

(28) Liu, D.; Xu, W.; Xiong, J.; He, L.; Zhao, Z. Electrochemical system with $LiMn_2O_4$ porous electrode for lithium recovery and its kinetics. *Separation and Purification Technology* **2021**, *270*, 118809-118817. DOI: 10.1016/j.seppur.2021.118809.

(29) Zhang, Z.; Zhang, J.; Zhang, Z.; Du, X.; Hao, X.; An, X.; Guan, G.; Li, J.; Liu, Z. Cross-linked PVDF-b-PAA composite binder enhanced $LiMn2O_4$/C film based electrode for selective extraction of lithium from brine with a high Mg/Li ratio. *Separation and Purification Technology* **2023**, *316*, 123777-123787. DOI: 10.1016/j.seppur.2023.123777.

(30) Zhang, Z.; Du, X.; Wang, Q.; Gao, F.; Jin, T.; Hao, X.; Ma, P.; Li, J.; Guan, G. A scalable three-dimensional porous λ-$MnO_2$/rGO/Ca-alginate composite electroactive film with potential-responsive ion-pumping effect for selective recovery of lithium ions. *Separation and Purification Technology* **2021**, *259*, 118111-118119. DOI: 10.1016/j.seppur.2020.118111.

(31) Chen, Y.; Zhan, H.; Qiao, Y.; Qian, Z.; Lv, B.; Wu, Z.; Liu, Z. Facet dependent ion channel of iron phosphate for electrochemical lithium extraction. *Chemical Engineering Journal* **2023**, *477*. DOI: 10.1016/j.cej.2023.147136.

(32) Zhang, J.; Su, W.; Yi, B.; Guo, Y.; Deng, T.; Yu, X. A new strategy for the preparation of highly stable and high-capacity electrodes for green electrochemical extraction of lithium. *Chemical Engineering Journal* **2023**, *454*. DOI: 10.1016/j.cej.2022.140416.

(33) Shang, X.; Hu, B.; Nie, P.; Shi, W.; Hussain, T.; Liu, J. $LiNi_{0.5}Mn_{1.5}O_4$-based hybrid capacitive deionization for highly selective adsorption of lithium from brine. *Separation and Purification Technology* **2021**, *258*, 118009-118017.

DOI: 10.1016/j.seppur.2020.118009.

(34) Tan, G.; Wan, S.; Chen, J. J.; Yu, H. Q.; Yu, Y. Reduced Lattice Constant in Al-Doped $LiMn_2O_4$ Nanoparticles for Boosted Electrochemical Lithium Extraction. *Adv Mater* **2024**, *36* (14), e2310657-e2310666. DOI: 10.1002/adma.202310657.

(35) Li, J.; Han, L.; Wang, R.; Wang, T.; Pan, L.; Zhang, X.; Wang, C. Lithium extraction via capacitive deionization: $AlF_3$ coated $LiMn_2O_4$ spheres for enhanced performance. *Desalination* **2024**, *591*, 118035-118043. DOI: 10.1016/j.desal.2024.118035.

(36) Zhan, H.; Qiao, Y.; Qian, Z.; Li, J.; Wu, Z.; Hao, X.; Liu, Z. Manganese-based spinel adsorbents for lithium recovery from aqueous solutions by electrochemical technique. *Journal of Industrial and Engineering Chemistry* **2022**, *114*, 142-150. DOI: 10.1016/j.jiec.2022.07.003.

(37) Du, X.; Guan, G.; Li, X.; Jagadale, A. D.; Ma, X.; Wang, Z.; Hao, X.; Abudula, A. A novel electroactive λ-$MnO_2$/PPy/PSS core–shell nanorod coated electrode for selective recovery of lithium ions at low concentration. *Journal of Materials Chemistry A* **2016**, *4* (36), 13989-13996. DOI: 10.1039/c6ta05985f.

(38) Qiao, Y.; Ran, Y.; Qian, Z.; Zhao, B.; Li, C.; Liu, Z. Overcoming polarization and dissolution of manganese-based electrodes to enhance stability in electrochemical lithium extraction. *Chemical Engineering Journal* **2024**, *497*. DOI: 10.1016/j.cej.2024.155009.

(39) Kim, S.; Kim, J.; Kim, S.; Lee, J.; Yoon, J. Electrochemical lithium recovery and organic pollutant removal from industrial wastewater of a battery recycling plant. *Environmental Science: Water Research & Technology* **2018**, *4* (2), 175-182. DOI: 10.1039/c7ew00454k.

(40) Liu, D. F.; Sun, S. Y.; Yu, J. G. Electrochemical and adsorption behaviour of $Li^+$, $Na^+$, $K^+$, $Ca^{2+}$, and $Mg^{2+}$ in $LiMn_2O_4$/λ-$MnO_2$ structures. *The Canadian Journal of Chemical Engineering* **2018**, *97* (S1), 1589-1595. DOI: 10.1002/cjce.23370.

(41) Zhao, Z.; Si, X.; Liu, X.; He, L.; Liang, X. Li extraction from high Mg/Li ratio brine with $LiFePO_4$/$FePO_4$ as electrode materials. *Hydrometallurgy* **2013**, *133*, 75-83. DOI: 10.1016/j.hydromet.2012.11.013.

(42) Wang, Q.; Du, X.; Gao, F.; Liu, F.; Liu, M.; Hao, X.; Tang, K.; Guan, G.; Abudula, A. A novel $H_{1.6}Mn_{1.6}O_4$/reduced graphene oxide composite film for selective electrochemical capturing lithium ions with low concentration. *Separation and Purification Technology* **2019**, *226*, 59-67. DOI: 10.1016/j.seppur.2019.05.082.

(43) Zhao, M.-Y.; Ji, Z.-Y.; Zhang, Y.-G.; Guo, Z.-Y.; Zhao, Y.-Y.; Liu, J.; Yuan, J.-S. Study on lithium extraction from brines based on $LiMn_2O_4$/$Li_{1-x}Mn_2O_4$ by electrochemical method. *Electrochimica Acta* **2017**, *252*, 350-361. DOI: 10.1016/j.electacta.2017.08.178.
